# Supplementary material for: Sub-nanometer mapping of strain-induced band structure variations in planar nanowire core-shell heterostructures
Source: Nat Commun. 2022 Jul 14;13:4089. doi: 10.1038/s41467-022-31778-3 (PMC9283334; doi:10.1038/s41467-022-31778-3)
Supplement: Supplementary file 1 — Supplementary Information [file 41467_2022_31778_MOESM1_ESM.pdf]

Supplementary Information

Sub-nanometer mapping of strain-induced band structure variations in planar nanowire core-shell heterostructures.

Sara Martí-Sánchez,[1,+] Marc Botifoll,[1,+] Eitan Oksenberg,[2] Christian Koch,[1] Carla Borja,[1] Maria Chiara Spadaro,[1] Valerio Di Giulio,[3] Quentin Ramasse,[4,5] F. Javier García de Abajo,[3,6] Ernesto Joselevich[2] and Jordi Arbiol[1,6,]*

1. Catalan Institute of Nanoscience and Nanotechnology (ICN2), CSIC and BIST, Campus UAB, Bellaterra, 08193 Barcelona, Catalonia, Spain
2. Department of Molecular Chemistry and Materials Science, Weizmann Institute of Science, Rehovot 76100, Israel
3. ICFO-Institut de Ciencies Fotoniques, The Barcelona Institute of Science and Technology, 08860 Castelldefels (Barcelona), Spain
4. SuperSTEM Laboratory, STFC Daresbury Campus, Daresbury WA4 4AD, United Kingdom
5. School of Chemical and Process Engineering & School of Physics and Astronomy, University of Leeds, Leeds LS2 9JT, United Kingdom
6. ICREA, Passeig Lluís Companys 23, 08010 Barcelona, Catalonia, Spain

[+]These authors equally contributed to the present manuscript

[*]Corresponding author: arbiol@icrea.cat

Table of contents

Supplementary Note 1. Growth directions, plane interactions and associated mismatches …………………………………………………………………. 3

Supplementary Note 2. Intermediate cases (gradual bending) ...……….………... 6

Supplementary Note 3. Strain tensor maps (ε_{ij})...…………………………………… 8

Supplementary Note 4. Atomic modelling on non-faceted cores ………………… 10

Supplementary Note 5. Additional details on strain relaxation mechanisms and shell rotation 24

Supplementary Note 6. Details on the Core-shell misfit dislocations ……………. 26

Supplementary Note 7. Simulations on VEEL spectra ………….............................. 28

Supplementary Note 8. Methodology for band gap mapping ……………………. 37

Supplementary References ….………………………………………………….... 51

Supplementary Note 1. Growth directions, plane interactions and associated mismatches

On C-plane (0001) sapphire the guided nanowires grow along six m $\pm\langle 1\bar{1}00\rangle$ directions, which reflect the three-fold symmetry of the plane and forming 60º between each growth direction (Supplementary Figure 1a-c). On A-plane $(11\bar{2}0)$ sapphire, the growth is produced along two m $\pm[1\bar{1}00]$ directions and four s directions $\pm[1\bar{1}01]$ and $\pm[1\bar{1}0\bar{1}]$. The m and s directions are separated by a 61.5° angle, and adjacent s directions are separated by a 57° angle.

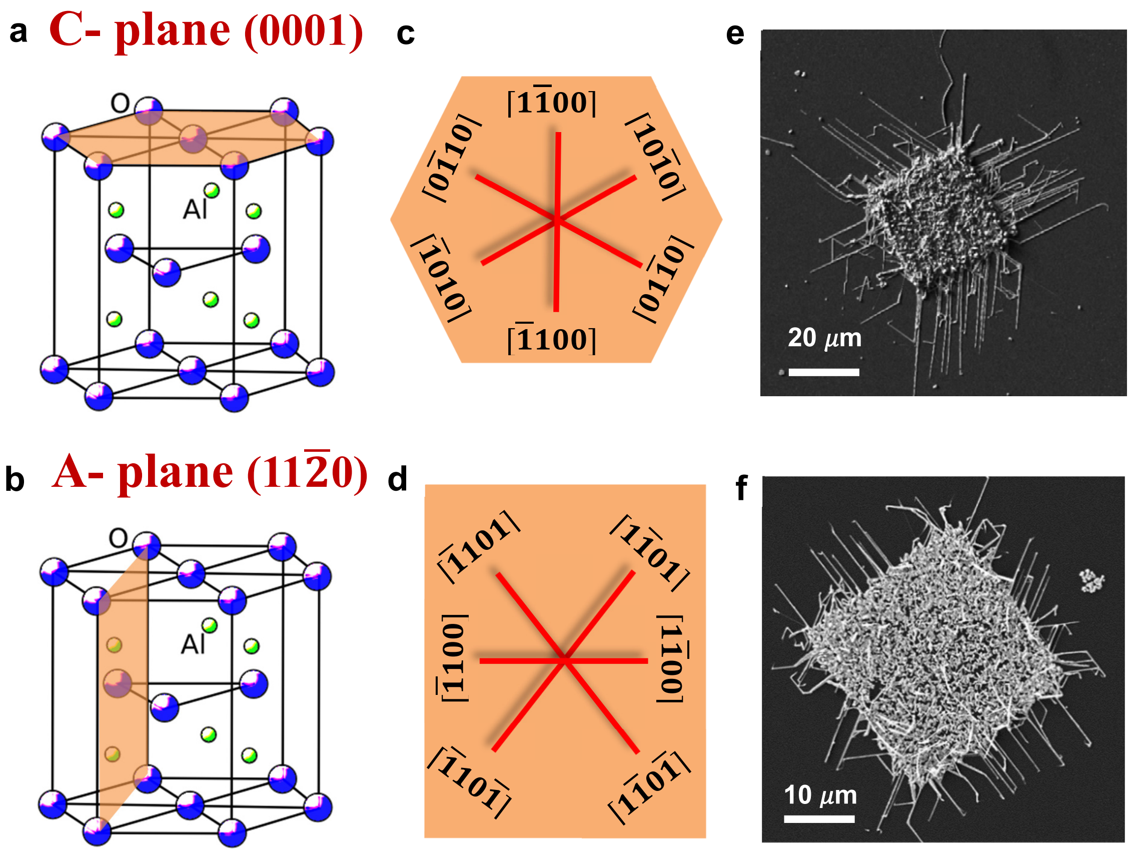

Supplementary Figure 1. a) and b) Schematics indicating sapphire A and C planes, respectively. c) and d) Nanowire growth directions in C- and A-plane oriented sapphire. e) and f) SEM micrographs showing a top view of nanowire arrays in C- and A-plane oriented sapphire, respectively.

Supplementary Figure 2 shows core morphologies associated to the growth along $[1\bar{1}00]$ direction on A- and C- plane sapphire surfaces.

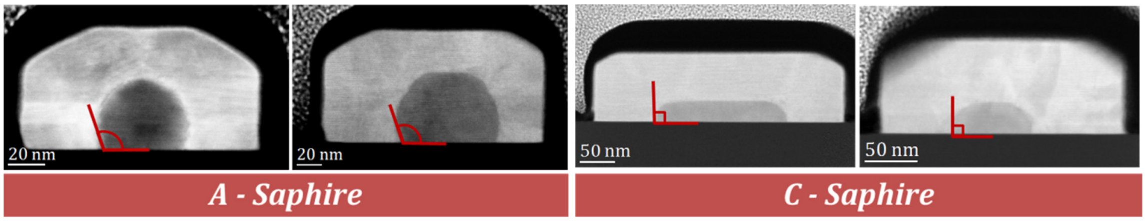

Supplementary Figure 2. Low magnification HAADF STEM micrographs obtained in cross-sections of nanowires growing along $[1\bar{1}00]$ sapphire directions in A-plane surface (left) and C-plane surface (right).

Lattice mismatch between substrate and nanowire is dependent on substrate orientation as the interacting planes are different in both cases.

Supplementary Table 1. Plane spacing list in ZnSe, ZnTe and α-Al_2O_3.

Material	Latice constants	{hk(i)l}	d_{hkl} (Å)
**ZnSe (cubic)**	a = 5.67 Å	111	3.27
		$1\bar{2}1$	2.32
		$20\bar{2}$	2.01
**ZnTe (cubic)**	a = 6.09 Å	111	3.52
		$1\bar{2}1$	2.49
		$20\bar{2}$	2.15
**α-Al_2O_3 (trigonal)**	a = 4.76 Å<br>c = 12.99 Å	0006	2.17
		$11\bar{2}0$	2.38
		$1\bar{1}00$	4.12
		$1\bar{1}02$	3.48

The mismatch between two materials can be calculated following Supplementary Equation 1.

$$\varepsilon\ (\%) = \frac{a_{substrate} - a_{material}}{a_{substrate}} \cdot 100$$ Supplementary Equation (1)

The calculated ZnSe - ZnTe mismatch is 7.4 %. The corresponding substrate – material epitaxial relations and mismatches are calculated from the plane spacing shown on Supplementary Table 1. They are listed in Supplementary Table 2.

In addition, in order to take into account the plastic relaxations and evaluate the remaining mismatch we used

$$\varepsilon'\ (\%) = \frac{m \cdot a_{substrate} - n \cdot a_{material}}{m \cdot a_{substrate}} \cdot 100$$ Supplementary Equation (2)

where a misfit dislocation occurs every *m* substrate planes and *n* material planes.

Supplementary Table 2. Epitaxial relationships between the substrate and ZnSe planes and associated plane mismatches.

Substrate orientation	Growth direction	Transversal direction	m : n	$\lvert\varepsilon'\rvert$ (%)
C-Sapphire	$[1\bar{1}00]Sapphire \vert\vert [1\bar{2}1]ZnSe$	$[11\bar{2}0]Sapphire \vert\vert [20\bar{2}]ZnSe$	1 : 1	15.6
			5 : 6	1.3
A-Sapphire	$[1\bar{1}00]Sapphire \vert\vert [1\bar{2}1]ZnSe$	$[0006]Sapphire \vert\vert [20\bar{2}]ZnSe$	1 : 1	7.4
			12 : 13	0.3
	$[1\bar{1}0\bar{1}]Sapphire \vert\vert [1\bar{2}1]ZnSe$	$[1\bar{1}02]Sapphire \vert\vert [20\bar{2}]ZnSe$	1 : 1	42.4
			2 : 3	13.6

Based on the calculated epitaxial relationships and associated mismatches in Supplementary Table 2, ZnSe nanowires grown following $\pm[1\bar{1}00]$ directions mainly relax strain by the creation of misfit dislocations. The residual strain is elastically accommodated within the first few nanometers of core growth, leading to a relaxed ZnSe lattice close to the junction.

Supplementary Note 2. Intermediate cases (gradual bending)

Different core morphologies have been found in the array samples apart from the most extreme cases shown in Figure 2 (main manuscript). Two examples presenting intermediate morphologies are shown in Supplementary Figure 3 for A- and C-plane sapphire-oriented samples. The core morphology of A-plane sapphire grown NWs tends to present rounded edges while in the case of C-plane sapphire the morphologies are more faceted.

The main difference in core morphologies between the two substrate orientations lies in the contact angle between ZnSe NW and substrate. While in the case of C-plane sapphire this angle is strictly 90º, for A-oriented sapphire the angle is always greater. This is attributed to different wetting angles of the catalytic droplet in the different sapphire surfaces.

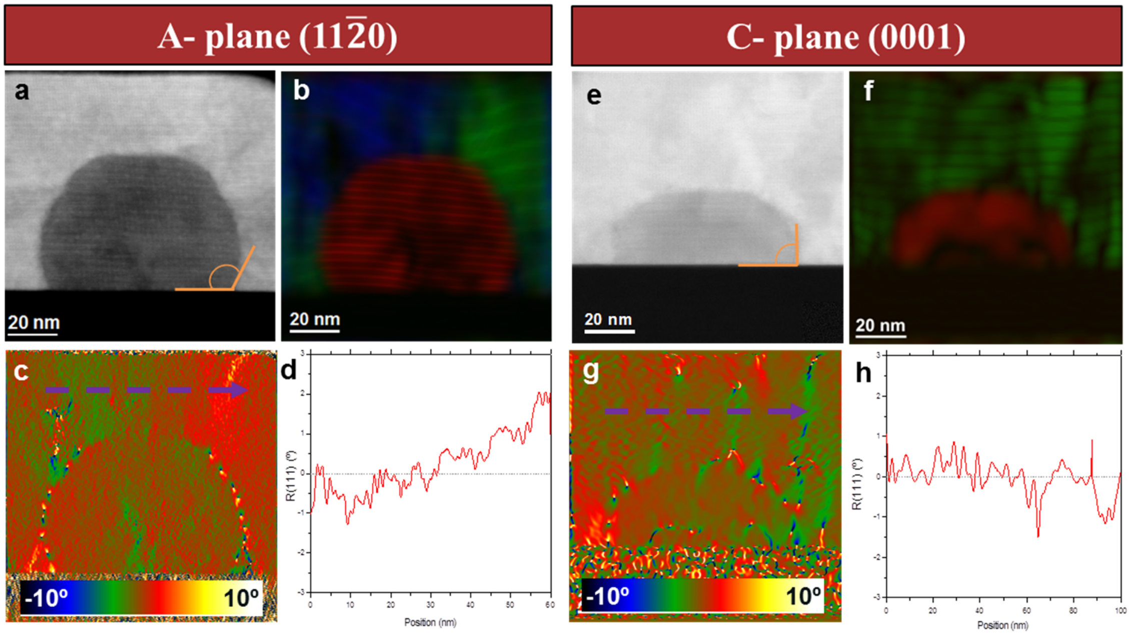

Supplementary Figure 3. Plane rotation analysis in intermediate cases. For A-plane sapphire, a) HAADF micrograph, b) color structural map, c) rotation map obtained through GPA applied to (111) planes and d) rotation profile obtained along the arrow indicated in c). For C-plane sapphire, e) HAADF micrograph, f) color structural map, g) rotation map obtained through GPA applied to (111) planes and h) rotation profile along the arrow indicated in g). Colour bars represent the geometrical phase analysis calculated (111) planes rotation in degrees (º).

Geometric Phase Analysis (GPA) applied to the (111) horizontal planes reveals a plane bending from -0.5º to +2º in A-oriented sapphire. However, the rotation inversion at both sides of the NW is gradual, so there is no appearance of a sharp boundary as in the purely cylindrical core presented in Figure 2 of the manuscript. In the case of C-plane sapphire NWs, no bending or nanowire sectioning is observed, with the intermediate case being similar to the one in flat belt-like nanowires.

Supplementary Note 3. Strain tensor maps (ε_{ij})

From the analyses of two orthogonal planes ((111) and (20$\bar{2}$)) we can obtain a full strain tensor component analysis (y≡[111], x≡[20$\bar{2}$]). Supplementary Figures 4 and 5 show the results applied to the two nanowires presented in Figure 2 of the main manuscript. The strain maps reveal no change in the unit cell volume, finding overall dilatations of 7.4 % and 7.3 % for ZnTe with respect to ZnSe in A- and C-plane sapphire, respectively. The discrepancy in the C-plane sapphire measurement with the expected 7.4 % associated to a relaxed lattice is within the range of error of the measurement. The relaxation is reached after 3 nm of shell growth in the case of the A-plane sapphire grown sample (cylindrical core) and after 7 nm in the case of C-plane sapphire grown NW (faceted core).

In both cases a distortion elongating horizontal planes and compressing vertical ones is observed, although it affects mainly cylindrical core nanowires.

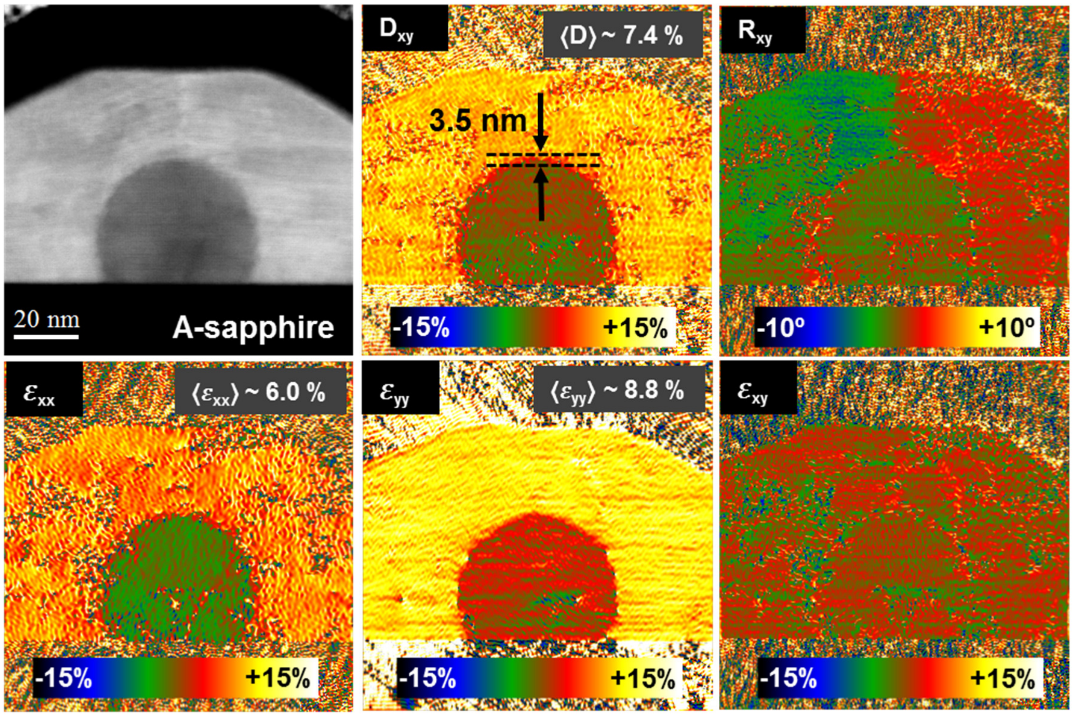

Supplementary Figure 4. Geometric Phase Analysis applied to the cylindrical core nanowire. Top: HAADF micrograph, overall dilatation and overall rotation maps. Bottom: strain matrix components ε_{xx}, ε_{yy} and ε_{xy} maps.

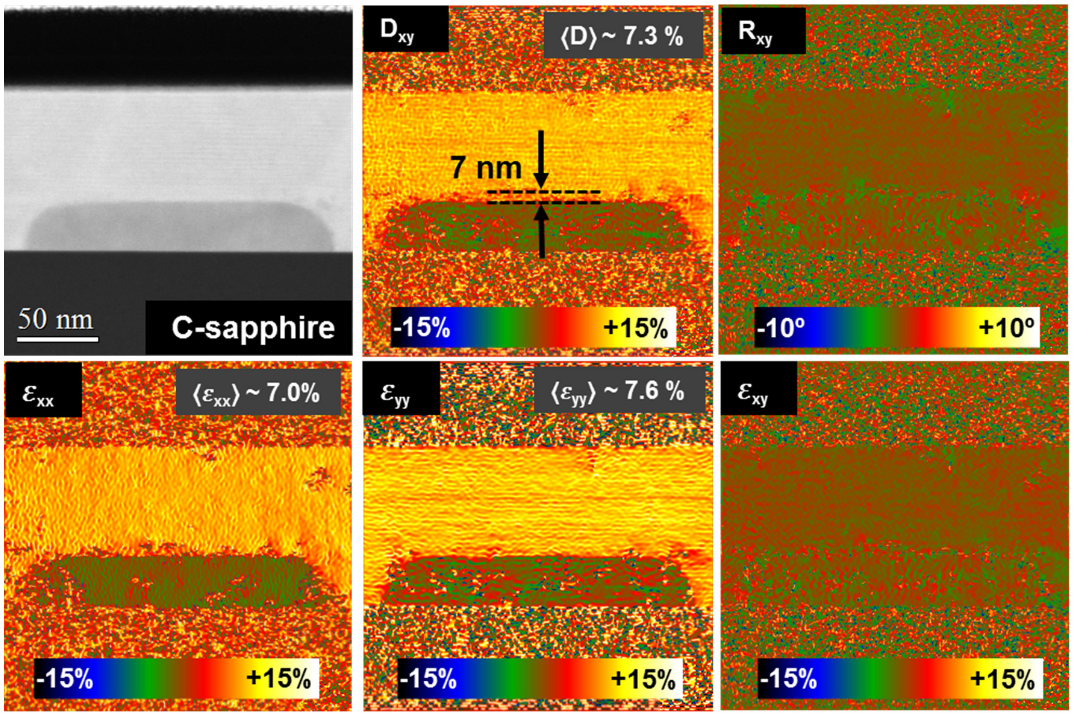

Supplementary Figure 5. Geometric Phase Analysis applied to the faceted core nanowire. Top: HAADF micrograph, overall dilatation and overall rotation maps. Bottom: strain matrix components ε_{xx}, ε_{yy} and ε_{xy} maps.

Supplementary Note 4. Atomic modelling on non-faceted cores

1st Approximation

The absence of bending in C-substrate nanowires can be attributed to the faceting strictly parallel to $(20\bar{2})$ planes. The effective interplanar distance of shell planes presenting a plane bending β interacting with the core is given by $d_{shell}^{eff} = d_{shell} / \cos\beta > d_{shell}$ Supplementary Equation (3). Considering that $d_{shell} > d_{core}$, the effective mismatch in a sharp interface increases with an increasing angle β.

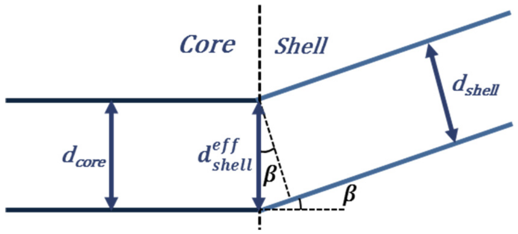

Supplementary Figure 6. Schematics of interplanar distances and angles when plane bending occurs.

The origin of plane bending in A-plane sapphire is related to the atomic steps originating from the curvature in nanosized geometry. For this reason, the closer to flat $(20\bar{2})$ surfaces, the lower bending angles minimize elastic energy accumulation.

STEM Simulations

Atomic models of the top left section of the nanowires have been created for relaxed core and shell lattices but inducing a crystal cell rotation around the nanowire growth axis ranging from 0º to 5º rotation, with steps of 1º. For each of them, the corresponding HAADF micrograph has been simulated reproducing the imaging conditions. Rotation maps of each micrograph have been obtained through GPA applied to (111) and $(20\bar{2})$ planes.

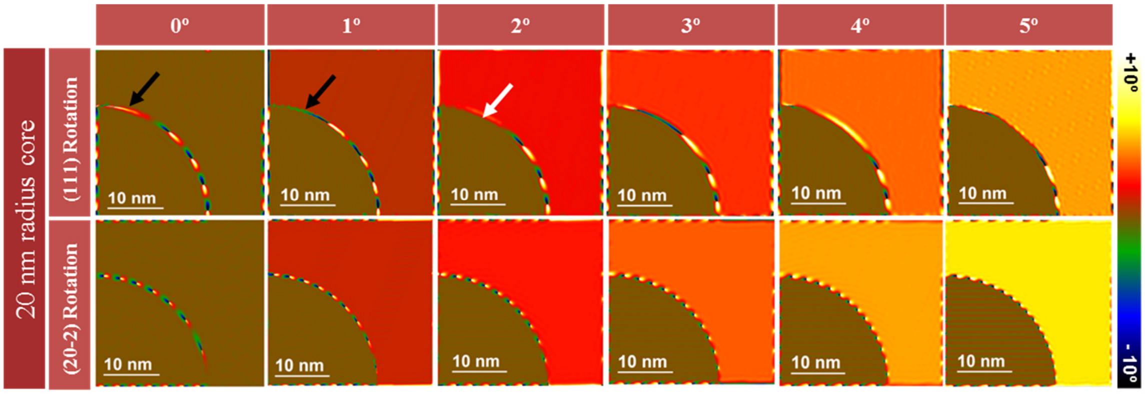

Supplementary Figure 7. Plane rotation obtained through GPA applied to (111) and $(20\bar{2})$ planes applied to atomic models with shell plane rotations ranging from 0º to 5º in order to evaluate the number of dislocations and plane matching.

Rotation maps of Figure 3 in the main manuscript visually reveal a minimization of elastic energy at the interface for a situation in which the shell crystal is rotated by 2º, as measured experimentally. We can consider the number of dislocations created by lattice mismatch as a way to quantify lattice adaptation, since an excess of elastic energy leads to the formation of dislocations (Supplementary Table 3). We can observe two different tendencies: for horizontal (111) planes, increasing rotation leads to a decrease in the number of misfit dislocations, while the opposite behaviour occurs in the case of vertical planes. The total number of dislocations formed in each case (directly related to the effective mismatch) increases substantially for angles higher than 3º). However, this tendency is not as clear for the cases in the range of 0 - 2º, where 1 dislocation is the maximum difference.

Observing the planes tangent to the cylinder ((111) in the top part and $(20\bar{2})$ in the right edge) we can evaluate their matching. A smooth accommodation of planes takes place in the case of 2º rotation (highlighted with a white arrow). However, a rugged interface is achieved in those regions for 0º and 1º (highlighted with black arrows). This reflects the worse accommodation of planes with lower bending angles. Elastic energy is accumulated in those areas despite no dislocation being fully formed because of the reduced size of the nanowire.

Therefore, the 2º situation measured experimentally minimizes the elastic energy of the system, even while creating anomalous grain boundaries at the central part of the nanowire.

Supplementary Table 3. Number of dislocations in horizontal and vertical planes for angles ranging from 0º to 4º in a 20 nm radius nanowire.

Rotation angle (º)	#(111) dislocations	#(20-2) dislocations	Total
**0**	4	7	11
**1**	4	8	12
**2**	3	9	12
**3**	4	11	15
**4**	2	13	15
**5**	2	15	17

Nanowire with 10 nm radius

The same analysis based on atomic modelling and HAADF STEM image simulations has been applied to a modelled nanowire with smaller radius (10 nm).

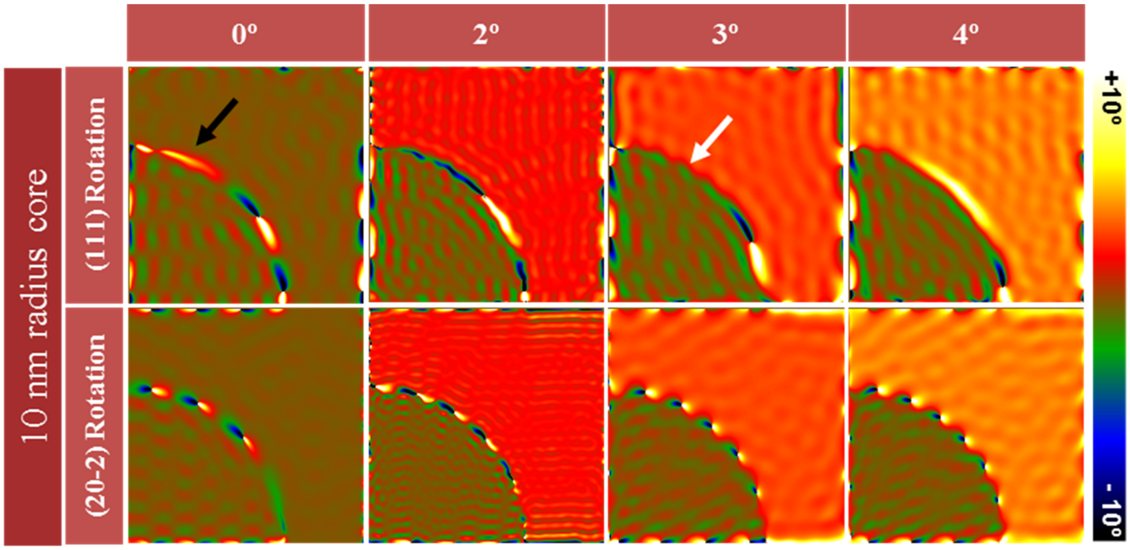

Supplementary Figure 8. Rotation maps on (111) and $(20\bar{2})$ planes obtained through Geometric Phase Analysis on an atomic model of the core-shell structure with 10 nm core radius.

When analysing the situation of a smaller core diameter (i. e. higher curvature), the induced rotation of 2º does not correspond to the most stable situation, which shifts to higher angles.

Taking into account the number of dislocations, as listed in Supplementary Table 4, a minimum is reached in the case of 0º and 3º. The matching between planes tangent to the interface is smoother with the 3º bending (highlighted with a white arrow), while elastic energy is accumulated without the formation of a misfit dislocation for the cases of 0º, 2º and 4º rotations. We conclude that a minimization of elastic energy is reached with plane rotations close to 3º for this core size.

Supplementary Table 4. Number of dislocations in horizontal and vertical planes for angles ranging from 0º to 4º in a 10 nm radius nanowire.

Rotation angle (º)	#(111) dislocations	#(20-2) dislocations	Total
**0**	2	4	6
**2**	2	5	7
**3**	1	5	6
**4**	1	6	7

Therefore, plane bending for elastic energy minimization in the case of higher curvature shifts to higher angles.

Nanowire core with 35 nm radius

From the conclusions obtained after analysing situations presenting small cores, we can predict that bending would tend to zero in the case of bigger core diameters. For this reason, the same methodology is applied to a core presenting a 35 nm radius.

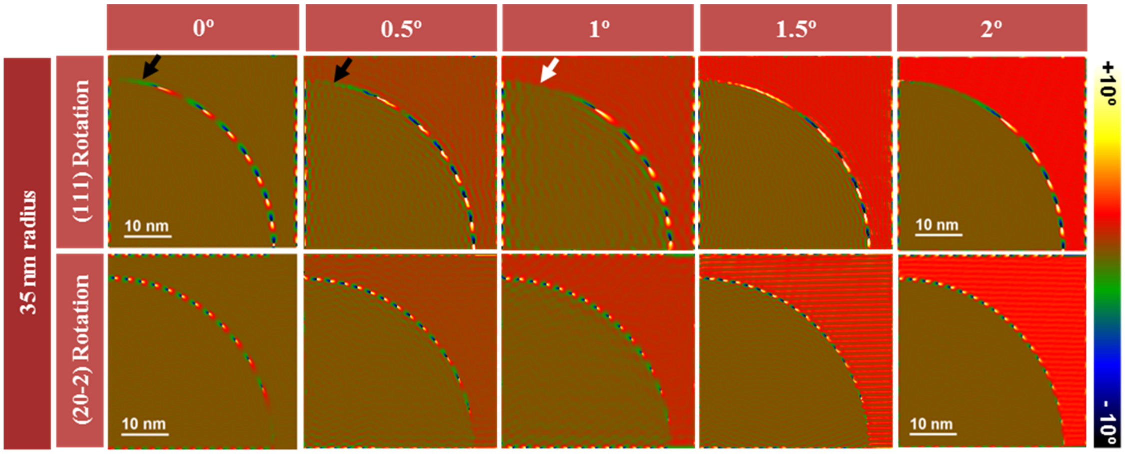

Supplementary Figure 9. Rotation maps on (111) and $(20\bar{2})$ planes obtained through Geometric Phase Analysis on an atomic model of the core-shell structure with 35 nm core radius.

As predicted, a better lattice accommodation is reached for lower bending angles. When analysing the total number of dislocations, as displayed in Supplementary Table 5, the minimal number of 20 dislocations is achieved with rotations ranging from 0 to 1º. Checking the matching of planes tangential to the core surface we find that 0º is far from optimal matching (black arrow), but the matching is improved for 1º bending in the case of (111) planes and 0.5º in the case of $(20\bar{2})$ planes. Elastic energy minimization is therefore assumed to happen between 0.5º and 1º. At the same time, we assume from this analysis that radii > 50 nm need to be achieved for equalling the flat plane situation of C-sapphire flat nanowires.

Supplementary Table 5. Number of dislocations in horizontal and vertical planes for angles ranging from 0º to 2 º in a 35nm radius nanowire.

Rotation angle (º)	#(111) dislocations	#(20-2) dislocations	Total
**0.0**	8	12	20
**0.5**	7	13	20
**1.0**	6	14	20
**1.5**	5	16	21
**2.0**	6	17	23

The analysis performed on different core radius diameters (i.e. different curvature) confirms the hypothesis that the main cause of the anomalous grain boundaries are the atomic steps caused by the curvature of the core. From the results obtained after modelling greater nanowire cores, the core dimensions should be greater than 70 nm to fully avoid plane bending, which would lead at the same time to a decrease in surface / volume ratio.

Controlling the wetting in surfaces for reaching contact angles of 90º appears as a more promising candidate, as plane rotations are avoided even for reduced NW core sizes.

2nd approximation

A second, more descriptive geometrical model has been developed to predict the position and number of misfit dislocations at the core-shell interface. This second approximation considers the effect of the core radius, r, and the angle the shell planes have with respect to those in the core.

In this model, we compute the number of dislocations per shell (n_d), given the radius of the core and the angle. Here we unveiled the details on how the non-linear variation of the effective distance between planes makes the dislocations arise, by computing the density of dislocations per length unit and validating the positions obtained by the atomistic simulations.

$$\rho_d = \frac{n_d}{\frac{2\pi r}{4}} \qquad \text{Supplementary Equation (4)}$$

Where ρ_d is the density of dislocations per perimeter unit.

Dislocations arise due to the different cell parameters, and consequently, different interplanar distances, in the substrates and in the materials grown on top. This scenario requires to define the mismatch differently, as no substrate is present, but a material next to another. Then, this mismatch m_s can be defined as follows, for a material 1 and 2:

$$m_s = \frac{a_1 - a_2}{a_2} \qquad \text{Supplementary Equation (5)}$$

With n and m being integers, the planes of the two materials match at the positions where the condition $na_1 = ma_2$ is fulfilled. Between two consecutive positions where the planes match, there will be one position where the planes of the two materials cannot be paired: the material with lower plane spacing will have a dangling plane without pair. These are the positions of the misfit dislocations.

First we set the distances and parameters involved in the system:

- The ZnSe core (specified as Se in the equations, being a_{Se} its cell parameter), with (hkl) = (111) planes perfectly horizontal and parallel to the substrate. Since no rotation is involved for these planes, each time we add a plane to the core we must just sum the interplanar distance, defined by:

$$d_{Se} = \frac{a_{Se}}{\sqrt{h^2+k^2+l^2}}\ for\ (111) \rightarrow\ d_{Se} = \frac{a_{Se}}{\sqrt{3}} \qquad \text{Supplementary Equation (6)}$$

- A different consideration applies to the ZnTe (specified as Te in the equations, a_{Te} being its cell parameter). Intuitively, at the very bottom, where the circular core shape begins, no curvature is observed, with only a continuous plane appearing. Nevertheless, if we start going up in height, the effect of the curvature becomes noticeable, and this infinite vertical plane becomes a bent curve that heavily influences the disposition of the atoms. Therefore, we define an effective distance for the ZnTe, d_{eff}, which is also related to its interplanar distance, $d_{Te}=a_{Te}/\sqrt{3}$.

Then, the condition that must be fulfilled for creating a dislocation becomes:

$$nd_{Se} = md_{eff}(\theta, y) \qquad \text{Supplementary Equation (7)}$$

where y is the height coordinate and we want to obtain the values for y at which the core and shell planes coincide. This can be done as both regions can be treated as mathematically independent from each other and just defined by the shape of their boundary. Of course, they are physically related, as the core defines how the shell grows, but we must think as if we could separate them both once having grown.

The definition of the increments in height defined in the core, due to the perfectly parallel condition is then very simple:

$$y(n) = nd_{Se} \qquad \text{Supplementary Equation (8)}$$

Next, we geometrically define the shell planes simulating the horizontal planes rotated θ degrees, to eventually use them to get the effective distance in contact with the core. To do this parametrization, the system in Supplementary Figure 10a, is taken as a reference. To define the linear function that describe the first shell plane, we use the coordinate (r,0) as its origin. In addition, as defined in the first approximation, the vertical projection of the planes is defined as p:

$$p = \frac{d_{Te}}{cos\theta} \qquad \text{Supplementary Equation (9)}$$

which is needed to compute the previously mentioned parametrization, as all the lines/planes of order m meet the condition of intersecting at (r, mp). The other parameter we need to fully characterize these lines is the slope, which is directly defined by the angle, θ:

$$y = ax + b \ \rightarrow a = tan\theta \qquad \text{Supplementary Equation (10)}$$

Then, for the first plane, order 0 (m=0):

$$y = tan\theta x + b \ \ intersecting \ (r, 0) \rightarrow 0 = rtan\theta + b \ ; \ b = -rtan\theta$$

Supplementary Equation (11)

$$Plane\ 0\text{:}\ y = tan\theta x - rtan\theta$$

For the second plane, order 1 (m=1):

$$y = tan\theta x + b \ \ intersecting \ \left(r, m\frac{d_{Te}}{cos\theta}\right) = \left(r, \frac{d_{Te}}{cos\theta}\right) \rightarrow \frac{d_{Te}}{cos\theta} = rtan\theta + b \ ;$$

Supplementary Equation (12)

$$b = \frac{d_{Te}}{cos\theta} - rtan\theta$$

$$Plane\ 1\colon\ y = tan\theta x + \frac{d_{Te}}{cos\theta} - rtan\theta$$

And, the mth order plane follows:

$$y = tan\theta x + b \ \ crosses \ \left(r, m\frac{d_{Te}}{cos\theta}\right) \rightarrow m\frac{d_{Te}}{cos\theta} = rtan\theta + b \ ; \ b = m\frac{d_{Te}}{cos\theta} - rtan\theta$$

Supplementary Equation (13)

$$Plane\ m\colon\ y = tan\theta x + m\frac{d_{Te}}{cos\theta} - rtan\theta$$

Note that we have defined that the first shell plane perfectly intercepts with the first geometric coordinate of the core. However, the most complete case would be to add an extra parameter with a value between 0 and the d_{eff} between plane m=0 and m=1, that would account for a possible shift in the growth of this first plane. Anyway, this addition would only slightly shift the obtained positions for the dislocations thought the perimeter. Nonetheless, it is important to keep its influence in mind, as it is the explanation for why some obtained dislocations are slightly displaced with respect to those in the atomistic models, together with the apparition or not of dislocations at the very bottom or very top of the core-shell interface.

With the previous parametrization, we have already considered the influence of the rotation angle in the model. To consider the effects of the curvature of the core, we must go one step further. Interestingly, these effects are clearly visible in Supplementary Figure 10b. The steps that are being created while we go to the top of the core vary a lot locally, and this is what we compute in the next and final step.

The key point here is that we must compute the direct intersection of the previously parametrised planes with the core circumference itself, defined in Supplementary Figure 10b, as the coordinates *c* and *e*. Keeping in mind the coordinate system, we initiated in Supplementary Figure 10.a, and the coordinates in b) we can define:

a(x,y)=(r,0)

b(x,y)=(r, $md_{Te}/cos\theta$*)=(r,* $d_{Te}/cos\theta$*)*

c(x,y) verifies:

$$y = \sqrt{r^2 - x^2} = tan\theta x + m\frac{d_{Te}}{cos\theta} - rtan\theta \qquad \text{Supplementary Equation (14)}$$

Strictly speaking, to get *c*, we should set *m*=1, but we can develop the previous equation in order to get to the general formulation:

$$\sqrt{r^2 - x^2} = tan\theta x + m\frac{d_{Te}}{cos\theta} - rtan\theta$$

$$r^2 - x^2 = tan^2\theta x^2 + \left(m\frac{d_{Te}}{cos\theta} - rtan\theta\right)^2 + 2tan\theta x\left(m\frac{d_{Te}}{cos\theta} - rtan\theta\right)$$

$$(tan^2\theta + 1)x^2 + 2tan\theta\left(m\frac{d_{Te}}{cos\theta} - rtan\theta\right)x + \left(m\frac{d_{Te}}{cos\theta} - rtan\theta\right)^2 - r^2 = 0$$

$$x = \frac{-2tan\theta\left(m\frac{d_{Te}}{cos\theta} - rtan\theta\right) \pm \sqrt{\left(2tan\theta\left(m\frac{d_{Te}}{cos\theta} - rtan\theta\right)\right)^2 - 4(tan^2\theta + 1)\left[\left(m\frac{d_{Te}}{cos\theta} - rtan\theta\right)^2 - r^2\right]}}{2(tan^2\theta + 1)}$$

$$x = \frac{-2tan\theta\left(m\frac{d_{Te}}{cos\theta} - rtan\theta\right) \pm \sqrt{-4\left(m\frac{d_{Te}}{cos\theta}\right)^2 + 8m\frac{d_{Te}}{cos\theta}rtan\theta + 4r^2}}{2(tan^2\theta + 1)}$$

Supplementary Equation (15)

By keeping only the positive solution, as the negative belongs to the diagonally opposed quadrant, we can get the abscissa of the intersection with plane *m*, x_m:

$$x_m = \frac{-tan\theta\left(m\frac{d_{Te}}{cos\theta} - rtan\theta\right) + \sqrt{r^2 + 2m\frac{d_{Te}}{cos\theta}rtan\theta - \left(m\frac{d_{Te}}{cos\theta}\right)^2}}{tan^2\theta + 1}$$

Supplementary Equation (16)

And the corresponding ordinate, y_m:

$$y_m = tan\theta x_m + m\frac{d_{Te}}{cos\theta} - rtan\theta$$ Supplementary Equation (17)

$$y_m = \frac{-tan^2\theta\left(m\frac{d_{Te}}{cos\theta} - rtan\theta\right) + tan\theta\sqrt{r^2 + 2m\frac{d_{Te}}{cos\theta}rtan\theta - \left(m\frac{d_{Te}}{cos\theta}\right)^2}}{tan^2\theta + 1} + m\frac{d_{Te}}{cos\theta} - rtan\theta$$

Supplementary Equation (18)

Summarising, the coordinates (x_m,y_m) indicating the intersection of the shell planes with the core circumference are computed, allowing us to calculate the effective increments the core sees from the shell.

In order to limit the values to study next, we can define the total number of planes in the core that will be needed to fill the entire core with (111) ZnSe planes:

$$\sum_{n=1,2,\ldots}^{n_{max}} d_{plans,nucli} = \sum_{n=1,2,\ldots}^{n_{max}} d_{Se} = r \to n_{max}d_{Se} = r \ ; \ \ n_{max} = \frac{r}{d_{Se}}$$

Supplementary Equation (19)

Likewise, for the shell, the m_{max} fulfils the following condition, which can be numerically solved:

$$r = \frac{-tan^2\theta\left(m_{max}\frac{d_{Te}}{cos\theta} - rtan\theta\right) + tan\theta\sqrt{r^2 + 2m_{max}\frac{d_{Te}}{cos\theta}rtan\theta - \left(m_{max}\frac{d_{Te}}{cos\theta}\right)^2}}{tan^2\theta + 1} + m_{max}\frac{d_{Te}}{cos\theta} - rtan\theta$$

Supplementary Equation (20)

Eventually, what we must do is compute all the ordinates, for each plane of order between 0 and n_{max} for the core, and between 0 and m_{max} for the shell, and compare them to identify those y that satisfies the condition $nd_{Se} = md_{eff}(\theta, y)$, and those that maximise the difference between coincident ordinates, i.e., dislocation positions. To do so, we compute the y at which the intersection with the core shape happens, and we correlate them with the absolute value of

the difference between the y_m of the shell and the y_n of the core = | $y_m - y_n$ |. The first perfect coincidence happens at y=0, as we have defined it in the model (that is why it is important to consider the influence of the previously mentioned shift parameter). This means that if we subtract the planes of same order $n = m$, we will find a | $y_m - y_n$ |=0 (i.e., intercept with the abscissa in absolute value functions), at $y = 0$. To find the next position of maximum coincidence, we must increase the order of the core planes compared to the shell, as the core has smaller projected distances in y. Then, when plotting $y = | y_m - y_{n+1} |$, we will obtain a 0 at the position of maximum coincidence between planes, meaning that between this minimum and the one computed before with m=n, y=0, we must find a dislocation. In fact, as can be seen in Supplementary Figures 10c-e plots, the nature of the absolute value gives us already the position of maximum plane discrepancy as well, which is of course, the dislocation itself. The same is done with $y= | y_m - y_{n+2} |$, $y= | y_m - y_{n+3} |$ and so on until the coincidence appears beyond the physical height of the core. The obtained positions and distribution of the dislocations match those obtained with the atomistic simulations at Supplementary Figure 7 very well. The cases of 0, 2 and 5 degrees are presented (Supplementary Figures 10c,d, and e respectively). Interestingly, the second approximation model not only reinforces the atomistic simulations, but it also provides an additional explanation to why the 2º scenario is the most stable one.

This explanation requires a detailed look at the plots in Supplementary Figure 10, together with the rotation maps in Supplementary Figure 7. The 0º case, which is represented in the plot as perfectly straight lines, in agreement with being equivalent to the first approximation, shows equally spaced positions for the perfect matches and mismatches. This is because the effective distance increase of this scenario is not affected at all by the curvature. In the 2º case, we see that the dislocation spacing is increasing as we go to the top of the core, and after the 3rd dislocation, the spacing of the shell planes becomes stabilised (i.e., varies only slightly) and

never reaches a | ym – yn |=0 condition again, meaning no new dislocation is generated. Since the formula we obtained is only affected by the angle and the curvature, we can be sure that this effect is only caused by the geometric effect that this exact rotation angle has on this exact core radius. This means, as we prove in the atomistic simulations with different radii, that the ideal rotation angle is solely defined by the core shape. On the other hand, with the 5º scenario, we observe an interesting effect caused by the excessive rotation compared to the ideal one.

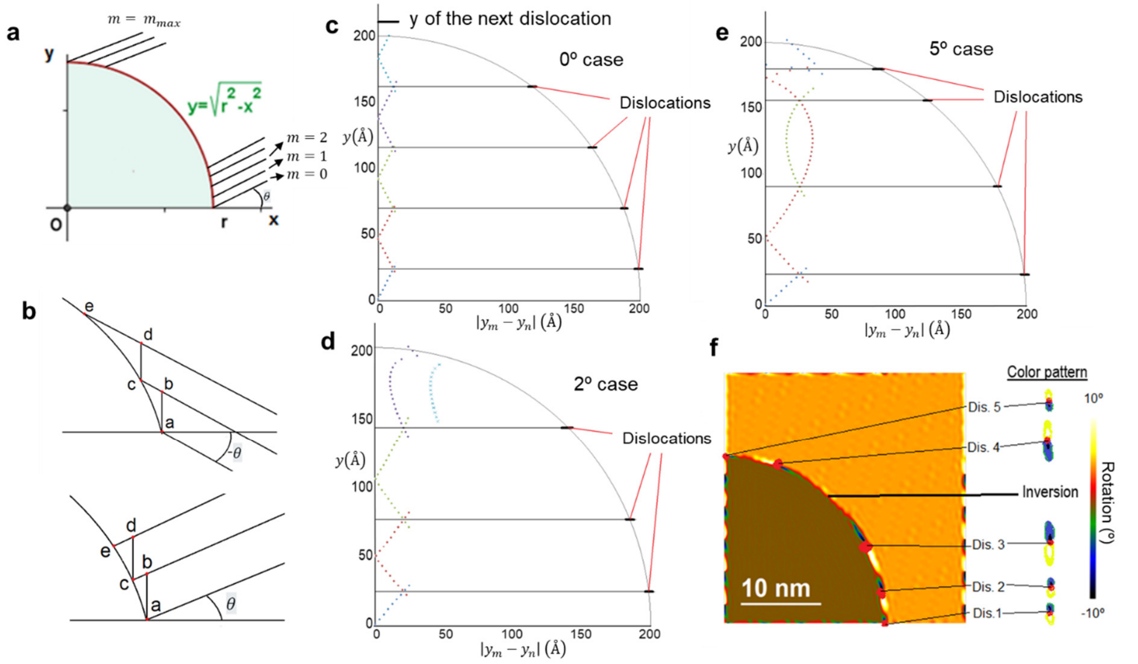

Supplementary Figure 10. (a) The 2D cartesian reference system used for parametrizing the shell planes and the effect of the curvature. (b) Detail on how the negative (positive) - graph above (below) - rotation of the shell planes affect their exact contacting position with the core. These coordinates are indicated with *c* and *e*, which are the key ones to be computed for properly considering the curvature effect. (c), (d) and (e), for the 0º, 2º and 5º bending of the shell respectively, plot the curves $y = | y_m - y_{n+k} |$, where each colour represents a different integer *k*. The intersections with the *y* axis display the points of maximum coincidence between planes, whereas the intersections of the curves give us the dislocation positions. The effect of the so-called dislocation inversion is seen in (e), where the red and green curve intersect in two coordinates, which is proved in (f) the 5º case atomistic simulation showing how the colour patterns of the dislocations are inverted due to the non-linear and excessive modification of the shell d_{eff} originated by the excessive rotation the 5º scenario implies.

This extra rotation has the effect that the otherwise stabilised effective distance of the shell in the 2º case now affects too much the variation of the shell effective distance, making the inversion of the usual tendency of the shell plane of one order below the core plane to meet the next planar coincidence, but return to meet the previous one that was met before. This is visible in the atomistic simulation for the 5º case, in Supplementary Figure 10.f, in which the colour pattern of the displayed dislocations is inverted due to this behaviour. Therefore, while the 2º case started to show this inversion pattern but never resulted in an extra dislocation due to the geometrical constraints, the overrotation of this simulated 5º case led to a different kind of dislocation that we call dislocation inversion, making it a less favourable scenario.

Supplementary Note 5. Additional details on strain relaxation mechanisms and shell rotation

It is important to note that the observed shell behaviour relates to the nanometric size of the core. First, for perfectly parallel core and shell atomic planes merging at the interface, we would expect a comparatively higher effective mismatch between both materials. However, if the atomic planes at the shell suffer elastic deformation and bend then the effective plane spacing of ZnTe at the interface is increased (Supplementary Figure 6). In addition, the small size of the rounded core cross-section renders its surface atomically stepped, resulting in high crystallographic matching when the effective interplanar distance of ZnTe is modified by the rotation of the shell lattice with respect to the core. Our atomic models created for nanowires presenting 10 nm core radius (higher curvature) and 35 nm core radius (lower curvature) show that there is also a core radius dependence on the optimal shell plane rotation angle for elastic energy minimization (Supplementary Figure 10). Therefore, the size of the catalyst droplet used during the VLS growth,[1] as well as its contact angle with the considered surface, play a key role in defining the core morphology and enabling the shell lattice to adapt to it. To model this behaviour, we developed a purely geometrical model that incorporates invariant interplanar distances in both ZnSe and ZnTe and accounts for the circular shape (of arbitrary radius) of the core, allowing the shell planes to rotate any angle around it. By forcing the model to fit the experimental radius of the core and different shell rotations (i.e.: 0, 2 and 5º), it captured the interplay between the curvature of the core and the optimal rotation angle of the shell required to minimize plastic deformations (misfit dislocations). The model supports a ±2º optimal rotation, given a 20 nm radius, also in agreement with atomistic simulations by explaining how the effective interplanar distance of the shell at the interface with the core is slightly varied along the curvature to avoid extra misfit dislocations. Larger rotation angles (e.g.: 5º) induce a variation of this effective distance that is too high and leads to the so-called dislocation

inversion phenomenon that generates additional plastic strain, as observed in Supplementary Figure 4 “2nd Approximation”.

Supplementary Note 6. Details on the Core-shell misfit dislocations

Our samples present two different types of misfit dislocations visible on our projected visualization axis:

1) On the lateral sides of the NW core-shell interface, misfit dislocations consist of the addition of a full (111) plane in the core with respect to the shell.

2) On the top side of the NW core-shell interface, misfit dislocations consist of the addition of half (10-1) plane in the core with respect to the shell.

Both types of defects are pure edge dislocations. In Supplementary Figures 11 and 12 we pointed the dislocation positions in GPA maps to help readers interpret their position in rotational maps and at the same time we have included their position and direction in the atomic resolution HAADF STEM images zooming them in detail. For clarity, we have applied a frequency filter to the HAADF STEM images.

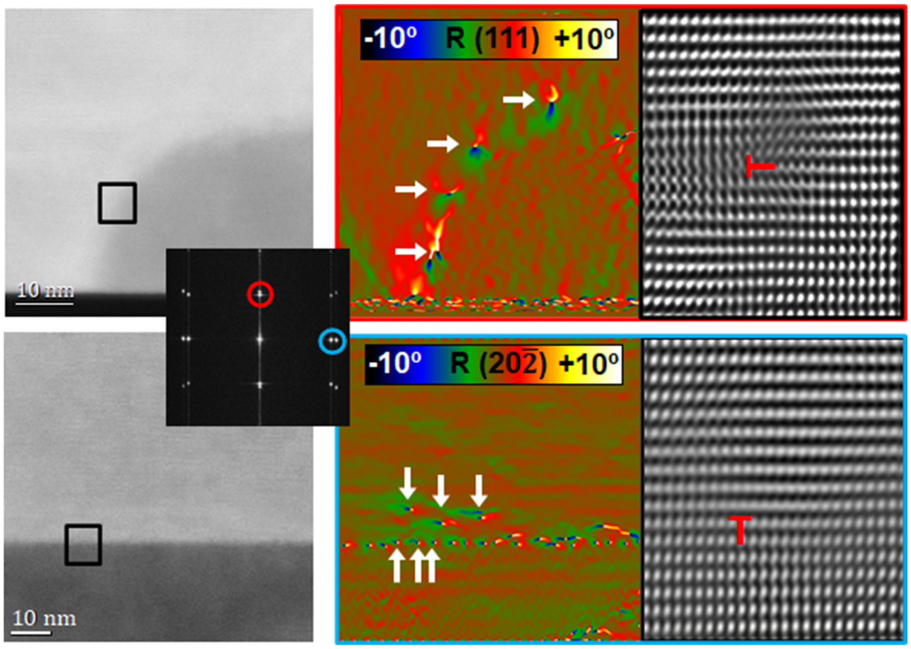

Supplementary Figure 11. Details of dislocation formation on (111) and (20-2) planes on a C-plane NW core-shell lateral and top interfaces, respectively. HAADF STEM micrographs zooming the core-shell interface, GPA rotation maps of (111) and (20-2) planes with arrows

indicating dislocation positions and zoom in of FFT plane filtered micrograph in the region marked in the HAADF STEM images indicating the dislocation configuration.

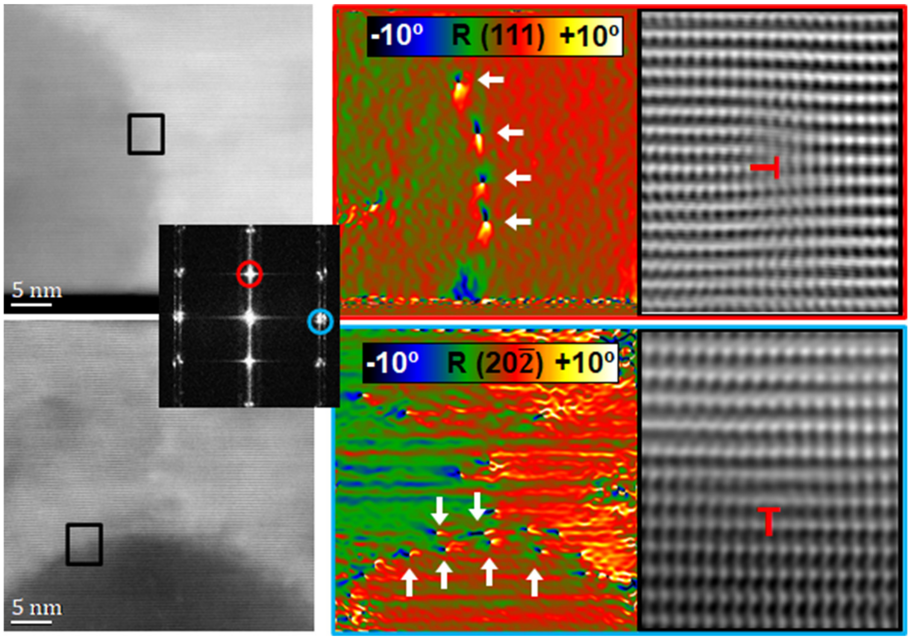

Supplementary Figure 12. Details of dislocation formation on (111) and (20-2) planes on a A-plane NW core-shell lateral and top interfaces, respectively. HAADF STEM micrographs zooming the core-shell interface, GPA rotation maps of (111) and (20-2) planes with arrows pointing interface dislocations and zoom in of FFT plane filtered micrograph in the region marked in the HAADF STEM images indicating the dislocation configurations.

Supplementary Note 7. Simulation of VEELS spectra

We have carried out STEM-VEELS simulations to study the influence of parasitic radiation contributing to the inelastic electron signal. Our simulations are based on classical electrodynamics, with the materials described by their frequency-dependent, local dielectric functions. The energy-loss probability is the sum of a bulk contribution, which is independent of geometry and proportional to the path travelled in each material, and a surface term determined by the interfaces and their distribution.[2] We have performed both retarded and non-retarded (i.e., assuming an infinite speed of light) calculations and compared them to assess the role of Cherenkov radiation emitted by the fast electrons in their interaction with the bulk materials. In fact, in this approach, the bulk contribution is rigorously emerging as that of an electron in an infinitely extended crystal.[3,4] This idea matches our experimental setup well, as the core-shell nanowire presents translational invariance along the direction of the electron beam over a relatively large distance. This allows us to compute the EELS probability per unit of electron path length, and eventually multiply this contribution by the length of the actual finite structures under examination (i.e., in TEM lamella carved through Focused Ion Beam (FIB)).

The bulk contribution including retardation is given by the expression[2,3]

$$\Gamma^{R}_{bulk}(\omega) = \frac{Le^2}{\pi\hbar v^2} Im\left\{\left(\frac{v^2}{c^2} - \frac{1}{\epsilon}\right) ln\left(\frac{{q_c}^2 - k^2\epsilon}{\left(\frac{\omega}{v}\right)^2 - k^2\epsilon}\right)\right\}, \qquad \text{Supplementary Equation (21)}$$

where

$$q_c \approx \frac{1}{\hbar}\sqrt{(mv\varphi_{out})^2 + \left(\frac{\hbar\omega}{v}\right)^2} \qquad \text{Supplementary Equation (22)}$$

is a momentum transfer cut-off defined by the collection semi-angle φ_{out}; and $k = \omega/c$ is the light wave vector in free space at the loss frequency ω; L is the thickness of the lamella; $\epsilon = \epsilon(\omega) = \epsilon_1(\omega) + i\epsilon_2(\omega)$ is the frequency-dependent dielectric function of the material at the beam position; v is the velocity of the incoming electron (v=0.446c at 60kV acceleration); and e is the electron charge.

In the non-retarded limit, this expression reduces to

$$\Gamma_{bulk}^{NR}(\omega) = \frac{2e^2L}{\pi\hbar v^2} Im\left\{-\frac{1}{\epsilon}\right\} \ln\left(\frac{q_c v}{\omega}\right), \qquad \text{Supplementary Equation (23)}$$

which does not account for Cherenkov losses. In fact, they are related to the denominator inside the logarithm in the retarded expression, the vanishing of which signals the dispersion relation of light inside the material.

We used the boundary-element method (BEM) to solve Maxwell's equations and obtain the energy-loss probability associated with interface corrections.[4,5] The average mesh size in the BEM simulations was around 2-3 parametrization points per nm along the perimeter of the boundaries, or equivalently, a separation of 0.3-0.5 nm between parametrization points. The computed range of energies was set to 0.5 eV to 10 eV. As in the bulk contributions, a translationally invariant system along the electron-beam direction was considered. Tabulated measurements of the local-response dielectric functions were employed for ZnTe,[6,7] ZnSe,[7,8] Al_2O_3[9,10] and Pt.[11] The latter was taken as an approximation of the protective metallic carbon matrix with platinum nanoparticles introduced during sample preparation by FIB. Given the large scale of the features in the nanowire (around 100 nm) we do not expect quantum confinement effects, which would modify the dielectric response of the involved materials, to play a significant role. Therefore, the bulk dielectric functions used as reference for the corrections should be suitable for our study. Incidentally, the BEM discretization used to obtain

converged simulations in commensurates with the spatial resolution of our measured spectral images. Therefore, atomistic effects such as dislocations are not considered within the simulated model used for correction of the experimental data. In fact, the effect of dislocations as low-band-gap centres adds up to the elastic strain to average the response that we observe in the band-gap maps. Atomistic effects could be eventually incorporated, for instance, via *ab initio* simulations similar to those employed in the context of atomic-resolution EELS spectral imaging.[12]

We applied the described methods to compute a spectral image consisting of 84x60 pixels in which the surface terms are obtained by incorporating a geometry deduced from the experimental images. We then obtained the total bulk distribution by adding the bulk contribution with or without retardation, as obtained from the expressions above with the dielectric function corresponding to each material at the beam position (i.e., $\Gamma_{total} = \Gamma_{bulk}^{R,NR} + \Gamma_{surf}$).

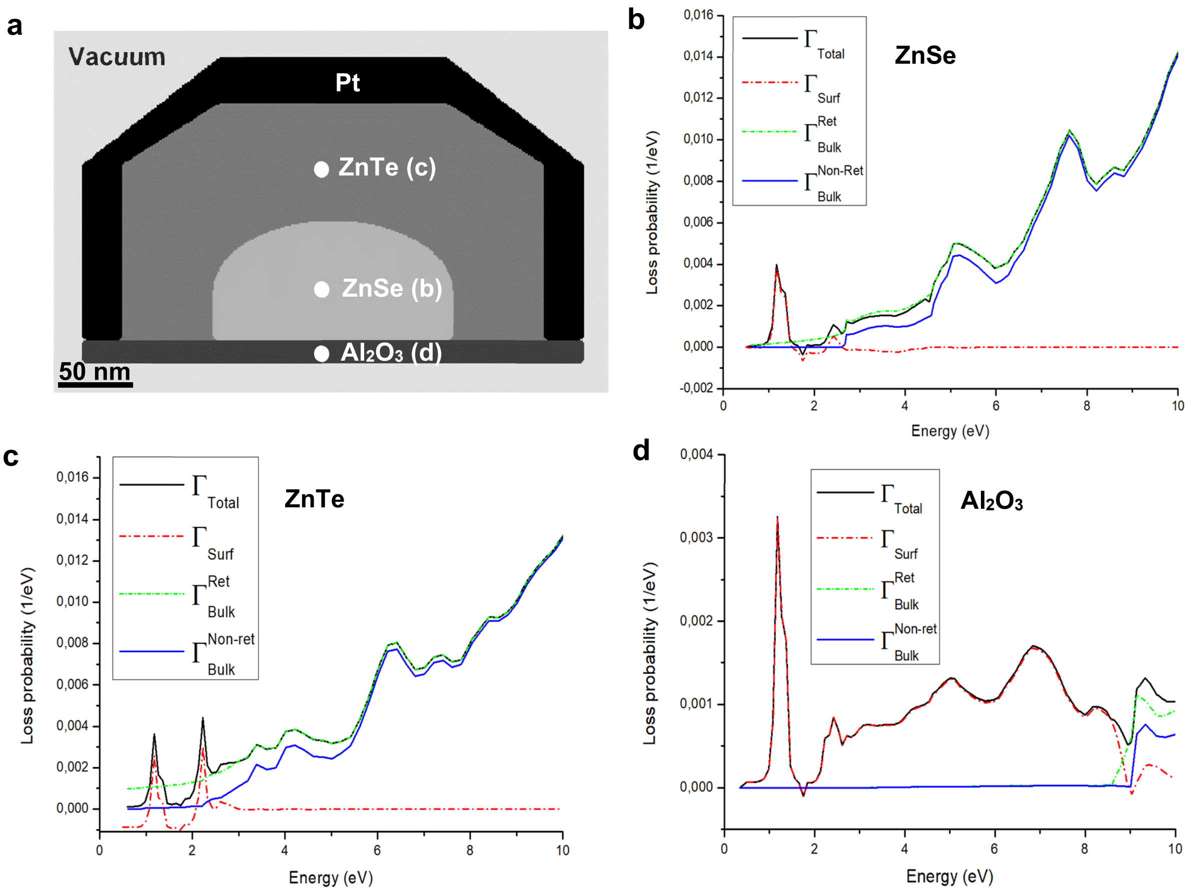

Supplementary Figure 13. (a) Geometry and homogeneous regions of the simulated spectral images. The different contributions are displayed in each plot: total signal (Γ_{Total}) in solid black; surface term(Γ_{Surf}) in dashed red; retarded bulk term (Γ_{bulk}^{R}) in dashed green; and non-retarded bulk term (Γ_{bulk}^{NR}) in solid blue, in good agreement with the experimental data. The white spots indicate a representative pixel to show its EELS probability for the main materials: (b) ZnSe, (c) ZnTe, and (d) Al_2O_3.

In the represented system, the main influence on the surface term is due to the cylindrical shape of the core and the shell. These geometries are reliably simulated within the BEM calculations, using the vertical and horizontal dimensions of both the core and the shell. In addition, the sapphire substrate and the model platinum coating have, in order to keep a computationally realistic grid, a region similar in size to the one observed in the spectral images, but as a result, smaller than the actual configuration of the system.

This thinner sapphire substrate induces the appearance of an intense waveguide mode peak centred at 1.17 eV that influences the signal that can be read in this energy region for the two main semiconductors. We proved that the emergence of this peak, of which there is no evidence in the experimental data, is due to the artificial addition of the extra interface (sapphire-air) when defining the sapphire substrate. Modification of the sapphire region dimensions revealed a variation in the position and width of this peak in the surface term of the loss probability, validating its origin. The presence or absence of this peak can be neglected as it is far away from the targeted energies in which signatures from electronic states should start to arise (>2 eV). Another feature that needs to be taken into account is the surface peak that appears in the ZnTe simulation, since it shows a different behaviour compared to the recorded data. This sharp peak, centred approximately at 2.1 eV, is broader and with the maximum shifted to the region between 2 eV and 2.3 eV in the experimental data. This is a possible source of error in the final result, particularly for retrieving the final band gap value of ZnTe (it could induce a variation smaller than 0.2 eV, which is within the pixelwise uncertainty of the method). These deviations are due to slight differences in the definition of the shapes of the ZnTe shell and the ZnSe core in the simulation when compared to the experimental shape, and the ensuing symmetry breakings that are obvious in the experimental spectral images. The computed EELS probability generally remains positive after summing the contributions from bulk and boundaries, except for a few points in the spectra without ever reaching large negative values. These may originate in the different treatment of the bulk and boundary components, since bulk losses have to incorporate a cut-off in transverse momentum transfers to avoid divergences and account for the finite range of the collected scattering angles, whereas such a cut-off is not included in the boundary contribution. In addition, the description of T-junctions and other sharp elements in the sample geometry add some imprecision to the numerical calculation because of their intrinsic singular nature. Nevertheless, we expect this effect to be minor at the

scale of the structures under consideration, as backed by the absence of any large negative values.

Strain considerations – The regions where the biggest changes in the band gap are expected are the surroundings of the interfaces, where the strain is located. The strain release mechanisms in this sample are fundamentally based on the creation of misfit dislocations and bending of the shell structure, which makes the resulting spatial distribution of the elastic deformation very localised and only spanning a few nanometres (between 3 nm and 7 nm depending on the radial position along the interface). The nature of the BEM simulations used does not take into account the possibility that strain affects the system, mainly by modifying the dielectric function of the materials at the interfaces. Although strain is not considered in the BEM simulations, an alternative experiment has been done to show that, given the expected strain, the used level of theory is enough to provide a trustworthy agreement with the experimental data. STEM-VEELS simulations have been performed to check whether an arbitrary variation in the band gap of ZnSe (ZnTe) due to strain, would affect the dielectric response (i.e., energy loss probability) of the ZnTe (ZnSe) in the first nanometres of the core-shell interface. For that purpose, the dielectric function of one of the materials is modified by making the material transparent to radiation at energies below approximately 5 eV (cancelling the imaginary part of the dielectric function), which simulates an enormous band gap deviation (increase) from the original conditions of the bulk material. While doing this, the other materials' properties are kept unchanged. These two variations capture all the possible scenarios of a hypothetical increase of either ZnTe or ZnSe band gap from its bulk reference value up to reaching a value of 5 eV. This is repeated by modifying the material left unchanged and keeping the original dielectric function of the first material. With this change and keeping the geometries and materials distribution exactly as in the main simulation, two new simulated data sets are obtained. The unmodified original simulation (the one used for the corrections)

can now be compared pairwise with each of the two new simulated data sets to map the deviations that the deliberate/artificial change in the band gap induced. The new simulated data is compared with the one obtained by the unmodified dielectric functions by performing a χ^2 analysis of the spectra in those pixels that surround the interface:

$$\chi^2(\boldsymbol{r}',\omega) = \frac{\left(\Gamma_{T,R}(\boldsymbol{r}',\omega) - \Gamma_{T,M}(\boldsymbol{r}',\omega)\right)^2}{\Gamma_{T,R}(\boldsymbol{r}',\omega)} \qquad \text{Supplementary Equation (24)}$$

where $\Gamma_{T,R}(\boldsymbol{r}',\omega)$ is the simulated total EELS signal with the unmodified/original dielectric functions at a certain position r' and energy ω; and $\Gamma_{T,M}(\boldsymbol{r}',\omega)$ is the simulated total EELS signal considering the modified dielectric function for one of the materials.

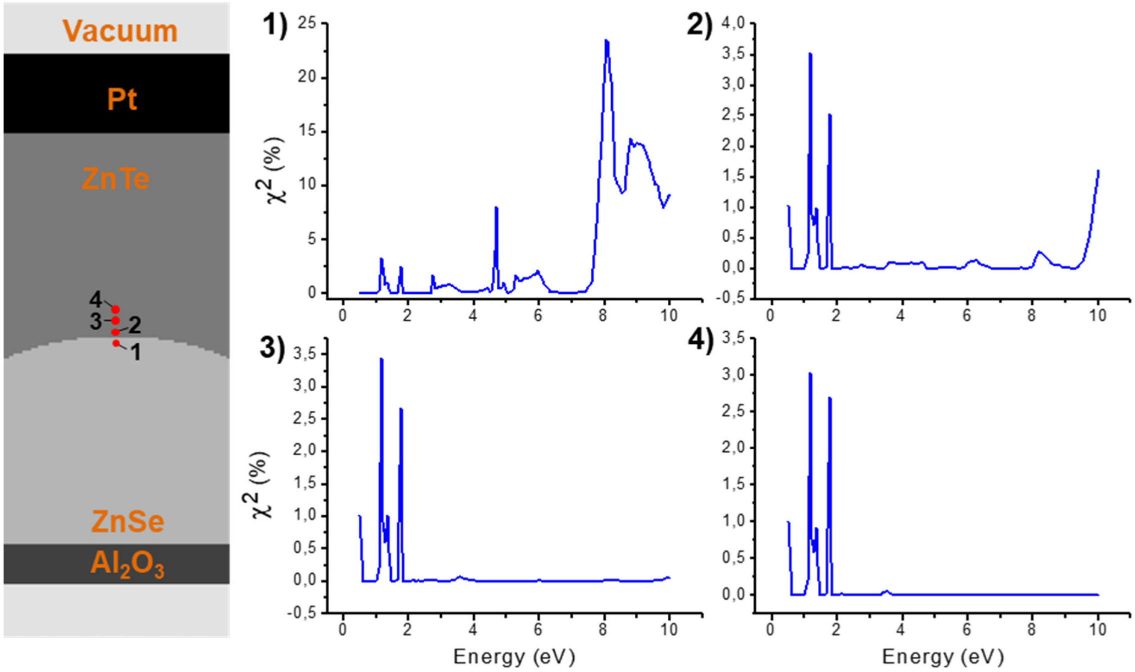

Supplementary Figure 14. χ^2 tests between unaltered simulations and ZnSe-shifted bandgap simulations to check the influence of ZnSe's bandgap modification on ZnTe's dielectric response. Four pixels are considered for this evaluation: the closest-to-the-interface ZnSe pixel (1), and the three ZnTe closest ones (2, 3 and 4). It shows that ZnTe would experience no dielectric deviation in the spectral region of interest (>2 eV) due to a hypothetical increase of the bandgap of the interfacial ZnSe.

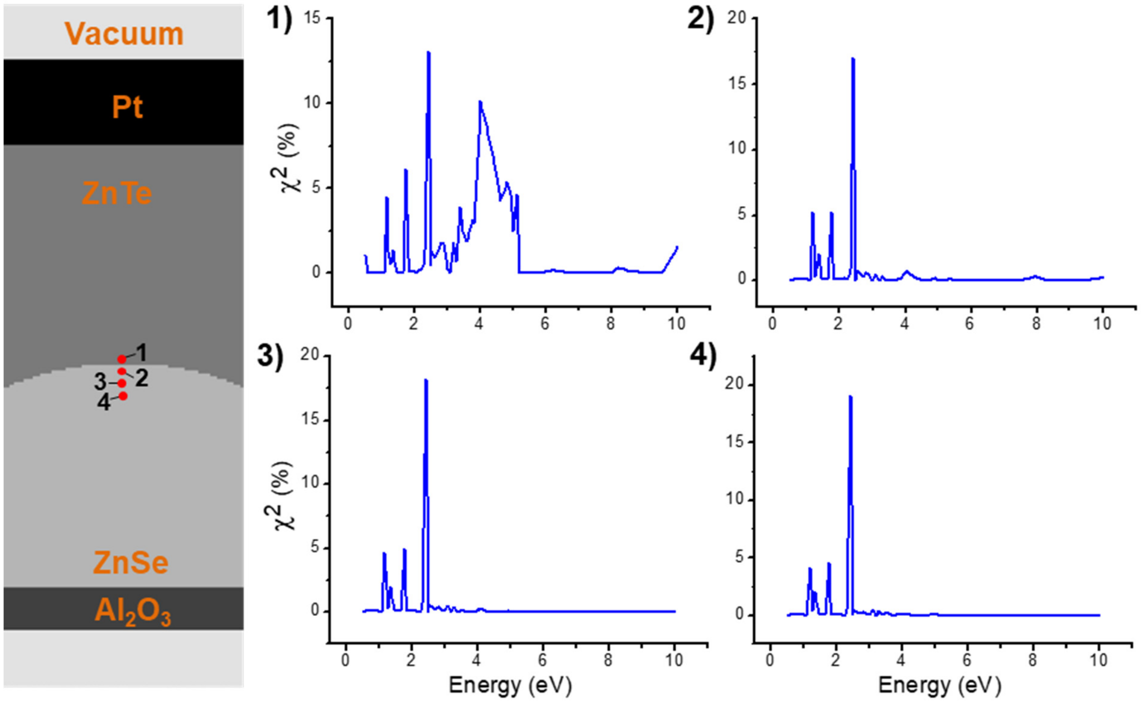

Supplementary Figure 15. χ^2 tests between unaltered simulations and ZnTe-shifted bandgap simulations to check the influence of ZnTe's bandgap modification on ZnSe's dielectric response. Four pixels are considered for this evaluation: the closest-to-the-interface ZnTe pixel (1), and the three ZnSe closest ones (2, 3 and 4). An important peak appears around 2.4 eV, meaning that a variation (increase) of ZnTe's bandgap would modify the loss probability of ZnSe in this specific energy region.

The results can be observed in Supplementary Figures 14 and 15. We have evaluated the effect that a change of the band gap energy in the shell would have at the core region near the interface and vice versa, by analysing vertical profiles crossing the core-shell interface. Supplementary Figure 14 evaluates the consequences of an increase in the band gap energy of the ZnSe core to the dielectric response of the nearby ZnTe shell. The null χ^2 curve at the targeted energies between 2 and 3 eV illustrates that no additional contributions to the low loss response are present when this hypothetical variation occurs. This means that the standard dielectric response would be the same as with a hypothetical increase in the band gap energy of ZnSe. On the other hand, Supplementary Figure 15 evaluates the influence of an increase in the band

gap energy of the ZnTe shell to the dielectric response of the ZnSe core region closer to the interface. In this case, the χ^2 curve is not zero through the spectral region of interest, meaning both the standard and modified responses would differ. Therefore, we will consider in our models that a hypothetic increase in ZnTe's band gap would induce the appearance of an extra peak around 2.4 eV in the core. This would happen because this peak corresponds to a waveguide mode in the shell, which it is strongly absorbed by the ZnTe in the real band gap scenario due to a nominal value of 2.26 eV (with the experimentally obtained average being 2.3162±0.0012 eV). Nonetheless, the forced band gap increase allows the radiation that was absorbed at the energies below the new band gap to propagate through the rest of the heterostructure, inducing therefore an extra contribution to the core. Despite this result, we do not expect or observe any increase in the ZnTe band gap, but rather a decrease due to compressive strain at the interface. This means that the 2.4 eV extra peak would be absorbed by the ZnTe itself regardless, without causing any undesired perturbation, which supports the validity of the obtained simulations and corrections.

Supplementary Note 8. Methodology for band gap mapping

The high energy resolution of around 150 meV (in vacuum zero-loss peak's (ZLP) full width at half maximum (FWHM), with a dispersion 50 meV/channel) provided by the Nion UltraSTEM^{TM} 100MC 'HERMES', at SuperSTEM (Daresbury, United Kingdom) and the dispersion of 50 meV/channel is reflected into a noisy (i.e. due to the performance of the energy-filtered and the intensity reduction by the monochromator) but recognizable definition of the local density of states (LDOS) of the involved materials. The absence of quantum confinement effects can be assumed given the feature's size of the device. Thus, a $(E\text{-}E_g)^{1/2}$ dependency should be expected for direct band gap materials, while a $(E\text{-}E_g)^{3/2}$ dependency must be considered for indirect band gaps in EELS.

In order to apply the data treatment routines explained below, the first step was to extract the spectrum-image to a format readable by Python3, the language in which the code has been developed. To do so, a DigitalMicrograph custom script is prepared to apply a generalized power law background model to the full spectrum image as a reliable tool for removing the ZLP. Moreover, each pixel has been smoothed down by a Savitzky-Golay filter with a second order polynomial, and the resulting data extracted as tabbed text.[13,14]

The main plasmon peaks of the ZnTe (centered at 15.3 eV), ZnSe (centered at 16.9 eV) and Al_2O_3 (centered at 25.4 eV) are used to map the local distribution of these materials. These intensity maps are thresholded to ensure they coincide with the domains observed in the spectrum-image, avoiding the effects of plasmon delocalization. Unsupervised machine learning has been used by means of Multivariate Statistical Analysis (MSA) to obtain component spatial maps of the interface pixels between ZnTe and ZnSe. We use it as a purely mathematical tool for locating the pixels in which we are interested, without initially assuming a physical relationship with the strain. Although it is not its physical origin, the spatial

distribution of the component agrees with the brightest regions observed in the MAADF-STEM image presented in Figure 5b, which highlights the regions containing strain accumulation (interfaces, volumetric defects…).

At this point, the use of the simulations becomes the critical step to get rid of the additional contributions to the energy loss spectrum, such as Cherenkov radiation and waveguide modes,[15,16] which can mask or complicate the extraction of the relevant band gap onset energy. The following considerations have been taken into account:

1) Every pixel in the experimental spectrum-image has been labelled with the material in which it was acquired, thanks to the thresholded mapping of their corresponding plasmons.

2) Every pixel in the simulated spectrum-image has been labelled with it the corresponding material. In addition, each pixel has its own calculation for each of the following components: total signal (Γ_{Total}), surface term (Γ_{Surf}), retarded bulk term (${\Gamma_{Bulk}}^{R}$) and non-retarded bulk term (${\Gamma_{Bulk}}^{NR}$).

For every pixel in the simulated spectrum-image, the following parameter called "Correction ratio", $\delta(\boldsymbol{r}', \omega)$, is computed per pixel:

$$Correction\,ratio = \delta(\boldsymbol{r}', \omega) = \frac{{\Gamma_{Bulk}}^{NR}}{\Gamma_{Total}} \;\; ; \; where \;\; \Gamma_{Total} = {\Gamma_{Bulk}}^{R} + \Gamma_{Surf}$$

Supplementary Equation (25)

This ratio is always a value between 0 and 1, and it represents the amount of signal or loss probability that comes intrinsically from the material at a given energy, ω, and position of the simulated spectrum-image, $\boldsymbol{r}'$, (in other words, signal free of all kinds of additional contributions), compared to the total signal or loss probability that also includes Cherenkov radiation and waveguide modes arising from the interfaces. Note that the correction ratio

involves loss probabilities that depend linearly on the thickness of the considered device. More precisely, the surface term obtained in the simulation is a loss probability per unit length along the thickness of the sample. Upon multiplication by the lamella thickness, each of the three terms in the ratio has the same linear dependence on thickness, thus making the ratio independent of this variable and robust against sample-to-sample thickness variations. In the present instance, the thickness was determined for ZnSe and ZnTe to have a uniform distribution around 45 nm. This is corroborated by the smooth thickness regions observed in Supplementary Figure 16, where variations in colour scale between materials are attributed to differences in the material-dependent inelastic mean free path, and not thickness changes. Moreover, the absolute thickness estimate, performed for the main materials ZnSe and ZnTe, revealed comparable average thicknesses of 44±3 nm and 46±1 nm, respectively.

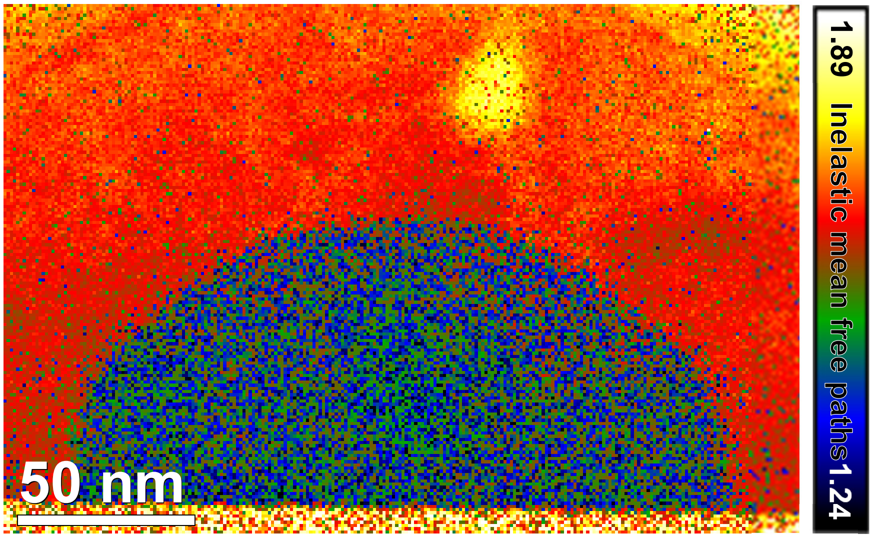

Supplementary Figure 16. Relative thickness map obtained by using the Digital Micrograph algorithm, in units of the inelastic mean free paths.

Next, each pixel from the experimental spectrum-image, defined by its material and position $\boldsymbol{r}$, is correlated to its representative pixel of the same material in the simulated spectrum-image, defined by $\boldsymbol{r}'$, and the following operation is performed to get the experimental EELS signal

with the accessible LDOS information, free of all the previously mentioned unwanted additional contributions, as Supplementary Figure 17 shows:

$$\Gamma_{Exp}{}^{Corrected}(\boldsymbol{r},\omega) = \delta(\boldsymbol{r}',\omega)\Gamma_{Exp}(\boldsymbol{r},\omega) \qquad \text{Supplementary Equation (26)}$$

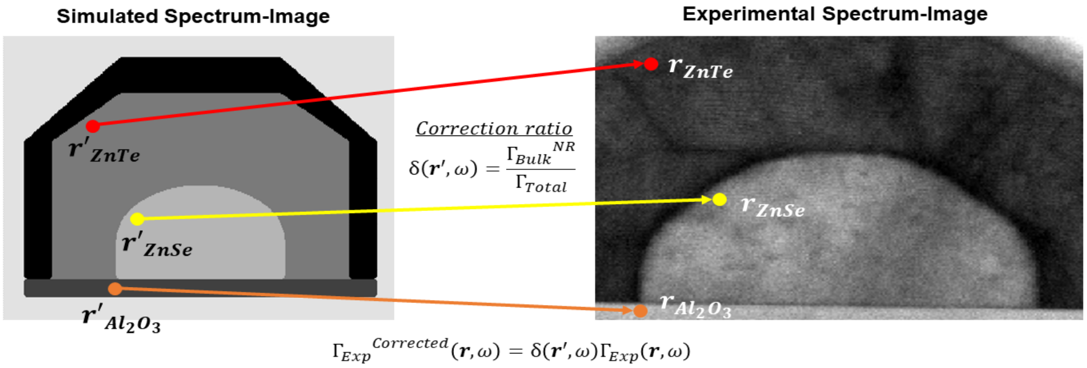

Supplementary Figure 17. Correction and relative position finding algorithm. For each material, every simulated pixel is linked to its representative experimental pixel (***r'→r***), and the correction ratio for that simulated pixel, ***r'***, is applied to the experimental data, ***r***, to obtain a new experimental spectrum-image free of Cherenkov and other additional losses.

A relative position finding algorithm is used to solve the variations in shape that the experimental spectrum-image has when compared to the ideal shape of the simulation. It allows to correlate the simulated and experimental positions (i.e., ***r'→r***) so that the curves in the shell and core are correlated between both spectrum-images. 1) For the core, we first find the relative horizontal position by normalizing the experimental position over the full length of the core. Given this relative position, the range of possible vertical values is found. For every horizontal position there is a different range of possible vertical values, which reaches its maximum exactly in the central pixels of the core (where the core is the highest) and its minimum on the left and right edges of the core. This relative vertical position as a function of the horizontal position is translated to the simulated spectrum-image in the same order: first we find the equivalent horizontal pixels, and between these, pick the one that fits the relative vertical position to be the final result, ***r'***. 2) The process in the shell is equivalent, but with minor

changes to adapt the symmetry. First, the shell is divided into three regions: pixels on top of the core, on its right and on its left. Then, the same process of the core applies for each division but finding the relative vertical position first in this case, and then the horizontal ones. This division into several regions is made to ensure that the curvature is considered, as the vertical position finds the height at which to consider the radius, and the horizontal component computes the radius at that height. Despite finding a good agreement between both shapes, it can be seen in the final maps how the noisiest area correspond to the shell pixels located at, thinking in the core as a clock's top semicircle, 11 and 1 o'clock, where the deviation of the shapes is highest. 3) For the sapphire substrate, the process is a bit simpler, and just comparing the rectangular sections and setting the dimensions to lie between 0 and 1 in both horizontal and vertical directions already allow to find the proper equivalent position. The corrections are applied independently for each of the previously defined correction regions, all of which are labelled by a single and univocal material. Thus, we make sure that the interface pixels of both ZnTe and ZnSe are being corrected by the same simulated material with the corresponding simulated pixel of the simulated spectrum-image.

The corrections are able to remove the peaks that arise due to the presence of waveguide modes, as well as reducing the total signal by removing a percentage of bulk Cherenkov losses at all the energies. As a result, the DOS curves become distinguishable and the correction method of removing a given percentage of the signal allows to keep the nature of the acquired curves and be sensitive to local band gap changes. Examples comparing the signal before and after the correction process can be seen in Supplementary Figure 18: these demonstrate the effective removal of the surface loss probability in sapphire, of the waveguide mode immediately before the expected ZnSe band gap energy and the compensation for an overall decrease in the ZnTe signal due to the high contribution of bulk Cherenkov losses. Indeed, the correction procedure could be refined by the deconvolution of the simulated curves with the experimental for a better

adaption of the simulated features in the final corrected signal. This routine would be complementary to the current percentage-based routine and could be of great interest if finer spectrum images, such as atomically resolved EELS, were analysed.

With the spectrum-image corrected in this fashion, the fitting of the functions that model the DOS can be done reliably: 1) By fitting a $(E-E_g)^{1/2}$ function in the case of a direct band gap material and 2) by fitting a $(E-E_g)^{3/2}$ function in the case of an indirect band gap material. To do that, a fitting algorithm evaluates, for every pixel and its material, all the possible energy ranges within a range of values that is centered at the reported bulk band gap value of each material. This adaptative range analysis is applied to make the computational cost tractable, as well as to reduce the generation of outliers. A goodness-of-fit analysis is performed in order to identify the numerically optimal spectral position at which these band gap energy characteristic patterns are located. For this purpose and due to the properties of the dataset and data analysis, the statistical parameter used is the coefficient of determination, r^2, defined as:

$$r^2 = 1 - \frac{\sum_i^n (y_i - \hat{y}_i)^2}{\sum_i^n (y_i - \bar{y})^2} \qquad \text{Supplementary Equation (27)}$$

where y_i denotes each dependent variable's value (i.e. electron counts) of the considered segment of data, $\hat{y}_i$ is the fitted value of the model at the same independent variable's value, and $\bar{y}$ is the mean value of the dependent variables of the considered segment of data. The fitting capacity of the algorithm has been proven by the evaluation of individual pixels over the full spectrum-image, showing a good ability of finding the adequate curve, as can be seen in Supplementary Figure 18.

The band gap error is estimated by the following considerations. Given a curve of the form $Counts=A(E-E_g)^n$, where n=1/2 or 3/2 depending on the band gap type, we fit a curve like $Counts^{1/n}=AE-AE_g$, to work with a linear dependence on the energy. This allows us to fit a linear polynomial $y=mx+c$, where $y=Counts$, $m=A$, $x=E$, and $c=AE_g$. The condition to extract

the band gap energy from this fitting is that y=0, allowing us to compute $E_g=-c/m$. The regression coefficients are computed with a regular fitting algorithm, and their respective standard errors are computed with the following equations:

$$s_m = \sqrt{\frac{\sum_{i=1}^{n}(y_i-\bar{y})^2}{(n-2)\sum_{i=1}^{n}(x_i-\bar{x})^2}} \qquad \text{Supplementary Equation (28)}$$

$$s_c = \sqrt{\frac{\sum_{i=1}^{n}x_i^2\sum_{i=1}^{n}(y_i-\bar{y})^2}{n(n-2)\sum_{i=1}^{n}(x_i-\bar{x})^2}} \qquad \text{Supplementary Equation (29)}$$

And given the propagation of errors:

$$s_f = \sqrt{\sum_i^l\left(\frac{\partial f}{\partial x_i}s_i\right)^2} \rightarrow s_{E_{g,stat}} = \sqrt{\left(\frac{1}{m}s_c\right)^2+\left(\frac{c}{m^2}s_m\right)^2} \qquad \text{Supplementary Equation (30)}$$

Then, the final error associated with the measure of the band gap is the combination (i.e., square root of sum of squares) of the experimental error, defined by the FWHM of 150meV, and the statistical error computed as before. We have observed that our statistical error is approximately one order of magnitude higher than the experimental error, meaning that we can approximate the total error of the measure as the statistical error, $s_{Eg,stat}=s_{Eg}$.

The performed fittings, based on the literature, are the following: direct band gap for ZnSe and ZnTe, and indirect for Al_2O_3.[17,18,19] The strained ZnTe interface pixels (as identified by the MAADF STEM – MSA correlated component thresholding analysis described above) have been also fitted with the indirect gap type function, with the assumption that ZnTe could undergo a band-type transition under compressive strain.[20] Statistical tests ($t \rightarrow z$ since n, the number of pixels, is always huge) have been performed by comparing the average band gap energy values per region to prove the significance of the observed small changes between the

band gap values of 'bulk' and interfacial pixels. Moreover, statistical tests have been carried out with the coefficients that discriminate the band gap type in the fitting to further support the band gap type evaluation.

Regarding the average band gap energy values and standard error of the mean for each region ('bulk' ZnTe pixels 2.3162±0.0012 eV ; interface ZnTe pixels = 2.28±0.01 eV ; interface ZnSe pixels = 2.755±0.011 eV ; 'bulk' ZnSe = 2.7733±0.0012 eV ; α-Al_2O_3 = 8.271±0.016 eV), there is a slight decrease of the band gap values of the interfaces in both materials (order of tens of meV), that is still significant given the large number of pixels ($\sim 10^3$ at the interface region and $>2 \cdot 10^4$ at the bulk region) used for its calculation. Interestingly, this is in good agreement with the reported effects calculated from theory in the band structure of ZnTe (ZnSe) under compressive (tensile) strain.[2020]

Furthermore, the indirect fitting performs significantly better than the direct one at the ZnTe interface pixels. As a control case, the same comparison of fitting coefficients is done with the bulk ZnTe pixels. Fitting an indirect gap in these pixels appears to result in a better goodness of fit as well, compared to a direct gap fit. However, in this case, the increase in the r^2 parameter can be solely attributed to an unphysical fit of the indirect curve to the tails of the waveguide mode clearly visible around 2.3eV, which give a much smaller and more disperse (2.1±0.3eV) result than the direct one. All the r^2 values, and the corresponding errors of the measurement, can be consulted at the final part of this supplementary note, "Significance tests". The worse performance of the direct curve fit in pure statistical terms (i.e., r^2) can be explained by the strong effect that the aforementioned waveguide mode and the pre-correction basal bulk Cherenkov radiation have around these energies, which produce an uneven curve making the fitting difficult. It is important to mention that due to waveguide modes being peaks, the sources of differences between the experimental system and the simulated one (i.e., slight geometry changes, assumed translational invariance and local thickness variations, delocalisation…)

make these modes the most challenging to identify. Hence the residuals of the tails of the most intense and shifted peaks can remain in the final curves, although most of their contribution is erased by the corrections (Supplementary Figure 18). For instance, the convolution of the tails of the waveguide mode centred around 2.2 eV, visible in the ZnTe domain as in Supplementary Figures 18c and d, leads us to expect a higher fitting error, especially in the edges of the nanowire shell where the deviation between the simulated and experimental geometry is greater. Nonetheless, the resulting direct fittings are more uniform and result in a standard deviation almost half of that obtained of the indirect fitting. In addition, there is no physical mechanism in our core-shell nanowire that would explain an almost 200 meV drop of the ZnTe reference bulk band gap. Similarly, the direct fitting applied to the interface ZnTe pixels (shown to be significantly worse than the indirect) leads to an average band gap value of 2.6±0.3 eV, which we believe is too large a shift given the computed levels of strain. Therefore, these arguments support the band type transition of ZnTe under compressive strain that has been reported theoretically.[20]

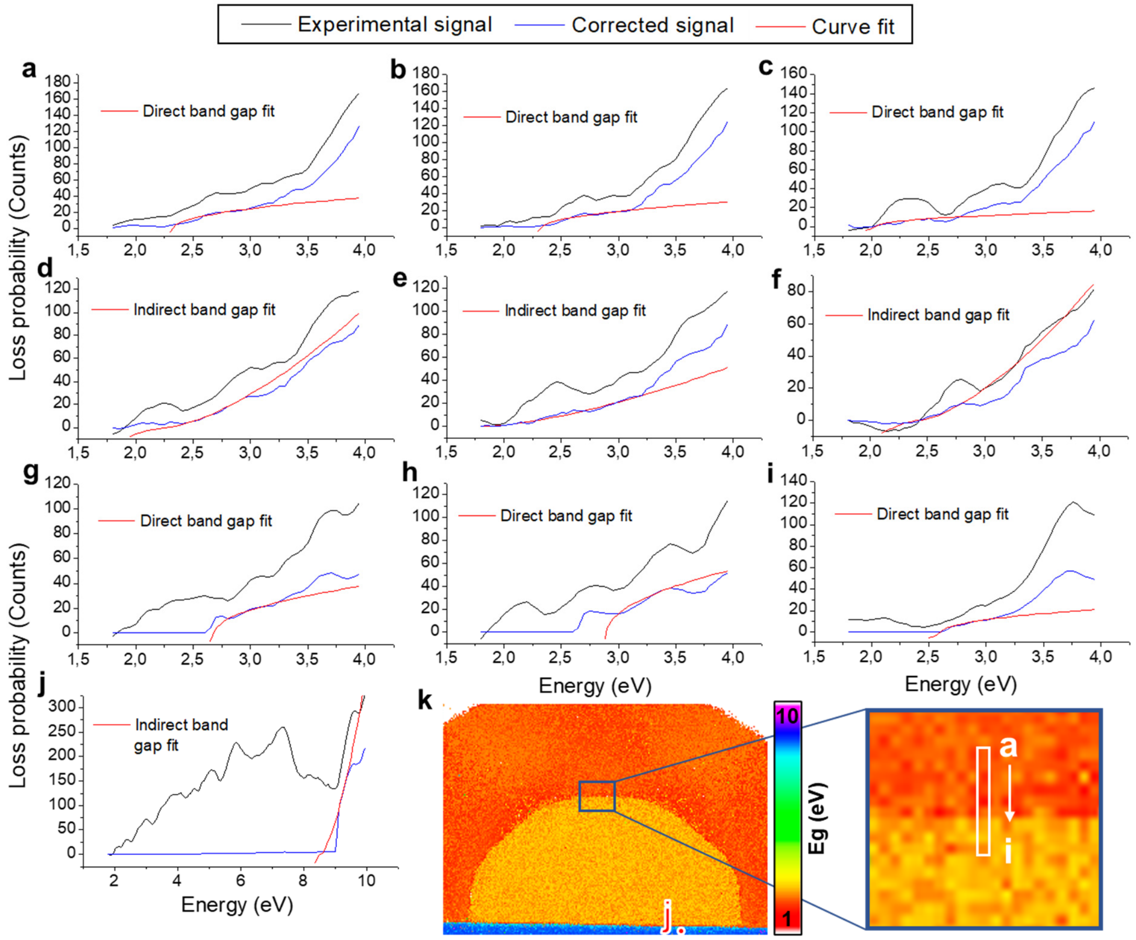

Supplementary Figure 18. Pixel-wise analysis of the correction method through a single pixel interfacial profile, indicated in the map and its zoom-in, k). a) to c) show ZnTe pixels and their direct curve fit; d) to f) show ZnTe interfacial pixels and their indirect curve fit; g) to i) show ZnSe pixels and their direct curve fit. j) shows an isolated sapphire pixel from its corresponding region, and its indirect curve fit.

Supplementary Figure 18 presents some of the most common scenarios encountered regarding the global performance of the method. The presence of local negative signal (counts) can be spotted in some individual, but only anecdotally and with no apparent localisation. Since the ZLP extraction follows the same power law model throughout the entire spectrum image, any artifacts causing a deviation from this general model in the corresponding spectral region can

result in a misfitted ZLP at a local level (pixelwise). Nevertheless, by studying the full spectrum-image we can confirm that this is not a common issue. Only a 1.59% of the total pixels, randomly distributed throughout the spectrum-image, had a 30% of channels with negative counts between 2 and 3 eV and with the average of these negative counts being larger in absolute terms than the 30% of the average of the remaining positive counts. Even when changing from 30% to 20% of channels in both considerations, which is much less harmful for the fitting, the value only rises to 4.69%. Moreover, this issue would require special caution in the percentage corresponding to ZnTe pixels, as the negative counts are typically centred around 2.17eV. Supplementary Figures 18a-c, show individual spectra from the ZnTe shell. The first two represent a good enough starting point regarding the quality of the experimental signal, which, after correction, leads to a quite accurate fitted function in the spectral range where the band gap lies (between 2 and 3 eV). Nevertheless, Supplementary Figure 18c shows how the waveguide mode at around 2.3 eV can make the correction troublesome and induce a shifted fitting. Supplementary Figures 18d-f are examples of ZnTe interface pixels. The main feature is the appearance of the indirect pattern close to the band gap energies, which is particularly clear in Supplementary Figure 18d. Supplementary Figure 18f displays the difficulty of choosing a pixel-wise perfect ZLP removing tool, and, despite working well overall, in some isolated pixels the background removal might be too aggressive, while in some other cases the removal may lead excessive residuals. In this case, this is translated into getting a higher-than-expected local band gap energy value. Supplementary Figures 18g-i are core ZnSe pixels, following the indicated profile. Here, the spectrum presents the remains of the DOS of the used reference dielectric function, visible as the abrupt increase of states around the ZnSe band gap energy. However, the fitting process still captures the essence of the experimental signal and allows to properly adapt the direct curve confirming this trend. Finally, Supplementary Figure 18j belongs to the sapphire region. Here we see how the surface modes

observed in Supplementary Figure 18d are completely removed and the curve indicating the real states becomes clear together with its indirect band structure fitting. Regarding curve fitting and the tolerance of the method to band-gap changes, the pixelwise evaluation of the fitted spectral ranges, revealed a trade-off between overfitting and noise handling at 1eV. Consequently, we expect that any band-gap shift of ±1 eV with respect to tabulated values to be properly captured by the model. Indeed, this range would be enough to resolve most of the phenomena that can induce band shifts. For larger band-gap shifts, it would be difficult to ensure the validity of the method that keeps the reference value as the one tabulated. In order to generalise this method and adapt it to larger variations, simulations should also consider band modulation by strain (e.g., from *ab initio* calculations).

Significance tests:

The following statistical tests have been performed to prove the statistical veracity of the statements we make throughout the article, in particular when comparing band gap values between regions, or when comparing different fit models. The values tabulated for the t distribution, which can be treated as a Gaussian given the number of samples used, are one-sided for the sake of our test purpose.[21]

Test 1: ZnTe interface pixels. Indirect fit vs Direct fit

Let us test if the indirect fit is better than the direct one in ZnTe interfacial pixels.

Null hypothesis: Both direct and indirect fits respond the same way: $H_0 \rightarrow \overline{r^2}_i = \overline{r^2}_d$

Alternative hypothesis: The indirect model fits the curve in the ZnTe interface pixels better than the direct model: $H_1 \rightarrow \overline{r^2}_i > \overline{r^2}_d$

Total number of ZnTe interface pixels = 904

Mean value of the r^2 coefficients for their indirect fit = 0.97191584

Standard deviation of the r^2 coefficients for indirect fit = 0.021585

Mean value of the r^2 coefficients for their direct fit = 0.9277318

Standard deviation of the r^2 coefficients for direct fit = 0.047758

$$t = \frac{\overline{r^2}_i - \overline{r^2}_d}{\sqrt{\frac{{s_{r^2}}_i^{\,2} + {s_{r^2}}_d^{\,2}}{n^2}}} = 762.12 \qquad \text{Supplementary Equation (31)}$$

The null hypothesis is rejected, and we can accept the alternative hypothesis with a confidence higher than 99.9995%: i.e., the indirect fit is significantly better than the direct one in ZnTe interface pixels.

<u>Test 2: ZnTe 'bulk' pixels. Indirect fit vs Direct fit (Control)</u>

Let us test if the indirect fit is better than the direct one in ZnTe bulk pixels.

<u>Null hypothesis:</u> Both direct and indirect fits respond the same way: $H_0 \rightarrow \overline{r^2}_i = \overline{r^2}_d$
<u>Alternative hypothesis:</u> The indirect model fits the curve in the ZnTe bulk pixelsbetter than the direct does: $H_1 \rightarrow \overline{r^2}_i > \overline{r^2}_d$
Total number of ZnTe bulk pixels = 22755
Mean value of the r^2 coefficients for their indirect fit = 0.951974713
Standard deviation of the r^2 coefficients for indirect fit = 0.05486646
Mean value of the r^2 coefficients for their direct fit = 0.85257474
Standard deviation of the r^2 coefficients for direct fit = 0.1427154

$$t = \frac{\overline{r^2}_i - \overline{r^2}_d}{\sqrt{\frac{{s_{r^2}}_i^{\,2} + {s_{r^2}}_d^{\,2}}{n^2}}} = 14793.1 \qquad \text{Supplementary Equation (32)}$$

The null hypothesis is rejected, and we can accept the alternative hypothesis with a confidence higher than 99.9995%: i.e., the indirect fit is significantly better than the direct one in ZnTe bulk pixels.

<u>Test 3: E_g ZnTe interface pixels (indirect) vs E_g ZnTe 'bulk' pixels (direct)</u>

Let us test if the band gap values obtained at the bulk pixels for ZnTe are bigger than the ones obtained for the interfacieal pixels of ZnTe (i.e., band gap reduction at the interface)

<u>Null hypothesis:</u> Both band gaps are the same statistically: $H_0 \rightarrow \overline{E_{g_b}} = \overline{E_{g_{int}}}$
<u>Alternative hypothesis:</u> The band gap in the bulk is statistically bigger than the interface band gap: $H_1 \rightarrow \overline{E_{g_b}} > \overline{E_{g_{int}}}$
Total number of ZnTe bulk pixels = 22755
Total number of ZnTe interfacial pixels = 904

Mean value of the band gap for ZnTe's bulk pixels = 2.316184

Standard deviation of the band gap for ZnTe's bulk pixels = 0.1865171

Mean value of the band gap for ZnTe's interfacial pixels = 2.281633

Standard deviation of the band gap for ZnTe's interfacial pixels = 0.291775

$$t = \frac{\overline{E_g}_b - \overline{E_g}_{int}}{\sqrt{\frac{s_{Eg_b}{}^2}{n_b{}^2} + \frac{s_{Eg_{int}}{}^2}{n_{int}{}^2}}} = 107.014 \qquad \text{Supplementary Equation (33)}$$

The null hypothesis is rejected, and we can accept the alternative hypothesis with a confidence higher than 99.9995%: i.e., there is a significant decrease in the band gap energy of the ZnTe interface compared to the bulk ZnTe.

Test 4: E_g ZnSe interface pixels vs E_g ZnSe 'bulk' pixels

Let us test if the band gap values obtained at the bulk pixels for ZnSe are bigger than the obtained in the interface pixels of ZnSe (i.e. band gap reduction at the interface)

Null hypothesis: Both band gaps are the same statistically: $H_0 \rightarrow \overline{E_g}_b = \overline{E_g}_{int}$

Alternative hypothesis: The band gap in the bulk is statistically bigger than the interface band gap: $H_1 \rightarrow \overline{E_g}_b > \overline{E_g}_{int}$

Total number of ZnSe bulk pixels = 14772

Total number of ZnSe interfacial pixels = 549

Mean value of the band gap for ZnSe's bulk pixels = 2.773333

Standard deviation of the band gap for ZnSe's bulk pixels = 0.14892

Mean value of the band gap for ZnSe's interfacial pixels = 2.7545514

Standard deviation of the band gap for ZnSe's interfacial pixels = 0.25151

$$t = \frac{\overline{E_g}_b - \overline{E_g}_{int}}{\sqrt{\frac{s_{Eg_b}{}^2}{n_b{}^2} + \frac{s_{Eg_{int}}{}^2}{n_{int}{}^2}}} = 40.987 \qquad \text{Supplementary Equation (34)}$$

The null hypothesis is rejected, and we can accept the alternative hypothesis with a confidence higher than 99.9995%: i.e., there is a significant decrease in the band gap energy of the ZnSe interface compared to the bulk ZnSe.

Supplementary References

[1] Zamani, M. et al. 3D Ordering at the Liquid–Solid Polar Interface of Nanowires. *Adv. Mater.* **32**, 2001030 (2020).

[2] Schattschneidera, P., Stoger, M., Hebert, C. & Jouffrey, B. The separation of surface and bulk contributions in ELNES spectra. *Ultramicroscopy* **93**, 91-97 (2002).

[3] García De Abajo, F. J. & Howie, A. Retarded field calculation of electron energy loss in inhomogeneous dielectrics. *Phys. Rev. B* **65**, 115418 (2002).

[4] García De Abajo F. J. Optical excitations in electron microscopy. *Rev. Mod. Phys.* **82**, 209-275 (2010).

[5] García De Abajo, F. J. & Howie, A. Relativistic Electron Energy Loss and Electron-Induced Photon Emission in Inhomogeneous Dielectrics. *Phys. Rev. Lett.* **80**, 5180 (1998).

[6] Talwar, D. N. & Becla, P. Polarization Dependent Reflectivity and Transmission for $Cd_{1-X}Zn_XTe$/GaAs (001) Epifilms in the Far-Infrared and Near-Infrared to Ultraviolet Region. *J. Material Sci. Eng.* **5**, 1000273 (2016). (Experimental results from 0.5eV to 2.5eV).

[7] Kootstra, F., de Boeij, P. L. & Snijders, J. G. Application of time-dependent density-functional theory to the dielectric function of various nonmetallic crystals. *Phys Rev. B* **62**, 7071 (2000). (Experimental results from 2.5eV to 10eV).

[8] Aven, M., Marple, D. T. F. & Segall, B. Some Electrical and Optical Properties of ZnSe. *J. Appl. Phys.* **32**, 2261 (1961). (Results from 0.5eV to 2.5eV).

[9] Tomiki, T. et al. Anisotropic Optical Spectra of α-Al2O3 Single Crystals in the Vacuum Ultraviolet Region. II Spectra of Optical Constants. *J. Phys. Soc. Jpn.* **62**, 1372-1387 (1993).

[10] Kasim Harman, A., Ninomiya, S. & Adachi, S. Optical constants of sapphire (α-Al2O3) single crystals. *J. Appl. Phys.* **76**, 8032 (1994).

[11] Werner W. S. M., Glantschnig K. & Ambrosch-Draxl, C. Optical Constants and Inelastic Electron-Scattering Data for 17 Elemental Metals. *J. Phys Chem Ref. Data* **38**, 1013-1092 (2009).

[12] Gutiérrez-Sosa, A. et al. Band-gap-related energies of threading dislocations and quantum wells in group-III nitride films as derived from electron energy loss spectroscopy. *Phys. Rev. B* **66**, 035302 (2002).

[13] Mitchell, D. R. G.'s script. Savitzky-Golay Spectral Filter for Digital Micrograph.

[14] Mitchell, D. R. G.'s script. Export Profile as Tabbed Text for Digital Micrograph.

[15] Stöger-Pollach, M. et al. Cerenkov losses: A limit for band gap determination and Kramers – Kronig analysis. *Micron* **37**, 396-402 (2006).

[16] Stöger-Pollach M. & Schattschneider P. The influence of relativistic energy losses on band gap determination using valence EELS. Ultramicroscopy 107, 1178–1185 (2007)

[17] Van Stryland, E. W., Woodall, M. A., Vanherzeele, H. & Soileau, M. J. Energy band-gap dependence of two-photon absorption. *Opt. Lett.* **10**, 490-492 (1985).

[18] Morkoç, H. et al. Large-band-gap SiC, III-V nitride, and II-VI ZnSe-based semiconductor device technologies. *J. App. Phys.* **76**, 1363 (1994).

[19] French, R. Electronic Band Structure of Al2O3 with Comparison to Alon and AlN. *J. Am. Ceram. Soc.* **13**, 471-489 (1990).

[20] Peköz, R. & Raty, J. Y. Band structure modulation of ZnSe/ZnTe nanowires under strain. *Phys. Rev. B* **84**, 1-8 (2011).

[21] STDH: Stadistical tools for high-throughput data analysis: t distribution table: (on line) <http://www.sthda.com/english/wiki/t-distribution-table>. Checked: 24th March, 2021.
